# Exploration of Nutraceutical Potentials of Isorhapontigenin, Oxyresveratrol and Pterostilbene: A Metabolomic Approach

**DOI:** 10.3390/ijms252011027

**Published:** 2024-10-14

**Authors:** Yu Dai, Jingbo Wang, Yuhui Yang, Hongrui Jin, Feng Liu, Hui Liu, Paul C. Ho, Hai-Shu Lin

**Affiliations:** 1 College of Pharmacy, Shenzhen Technology University, Shenzhen 518118, China; 2 Department of Pharmacy, National University of Singapore, 18 Science Drive 4, Singapore 117543, Singapore; 3 Institute of Materials Research and Engineering (IMRE), Agency for Science, Technology and Research (A*STAR), 2 Fusionopolis Way, Innovis #08-03, Singapore 138634, Singapore; 4 Quality and Standards Academy, Shenzhen Technology University, Shenzhen 518118, China; 5 School of Pharmacy, Monash University Malaysia, Jalan Lagoon Selatan, Bandar Sunway, Subang Jaya 47500, Malaysia

**Keywords:** resveratrol, isorhapontigenin, oxyresveratrol, pterostilbene, metabolomics

## Abstract

Resveratrol (*trans*-3,5,4′-trihydroxystilbene, RES) is one of the most well-known natural products with numerous health benefits. To explore the nutraceutical potentials of some dietary RES derivatives including isorhapontigenin (*trans*-3,5,4′-trihydroxy-3′-methoxystilbene, ISO), oxyresveratrol (*trans*-3,5,2′,4′-tetrahydroxystilbene, OXY) and pterostilbene (*trans*-3,5-dimethoxy-4′-hydroxystilbene, PTS), their impacts on metabolism and health were assessed in Sprague Dawley rats after a two-week daily oral administration at the dose of 100 µmol/kg/day. Non-targeted metabolomic analyses were carried out with the liver, heart, brain and plasma samples using gas chromatography–tandem mass spectrometry (GC-MS/MS). Notable in vivo health benefits were observed, as the rats received ISO, PTS or RES showed less body weight gain; the rats received OXY or RES displayed healthier fasting blood glucose levels; while all of the tested stilbenes exhibited cholesterol-lowering effects. Additionally, many important metabolic pathways such as glycolysis, pentose phosphate pathway, tricarboxylic acid cycle and fatty acid oxidation were found to be modulated by the tested stilbenes. Besides the reaffirmation of the well-known beneficial effects of RES in diabetes, obesity, cardiovascular disease and Alzheimer’s disease, the metabolomic analyses also suggest the anti-diabetic, cardio-, hepato- and neuro-protective activities of ISO; the anti-diabetic, cardio-, hepato- and neuro-protective effects of OXY; and the anti-aging, anti-inflammatory, cardio-, hepato- and neuro-protective potential of PTS. Interestingly, although these stilbenes share a similar structure, their biological activities appear to be distinct. In conclusion, similarly to RES, ISO, OXY and PTS have emerged as promising candidates for further nutraceutical development.

## 1. Introduction

**Figure 1:**
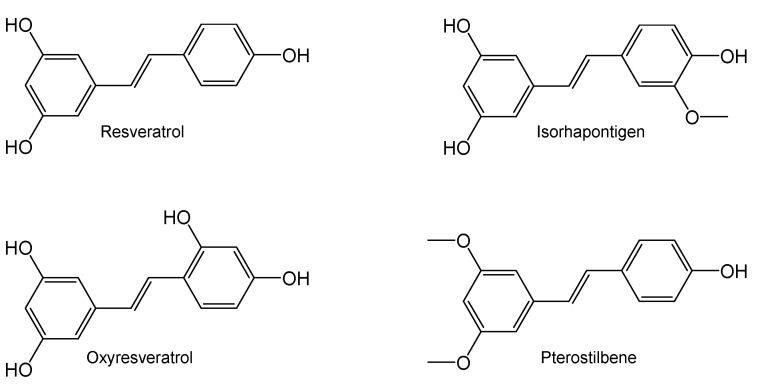
Chemical structure of resveratrol and its dietary derivatives.

Resveratrol (*trans*-3,5,4′-trihydroxystilbene, RES, Figure 1), one of the most well-known natural products, is a polyphenolic phytoalexin present in human diets by means of grapevine, red wine, cranberry, blueberry, bilberry and peanut [1,2]. During the past twenty years, its pleiotropic health benefits have attracted great attention in the biomedical research community [1,2]. The clinical efficacy of RES in various medical conditions, e.g., Alzheimer’s disease, cancer, cardiovascular diseases, chronic kidney disease, diabetes, nonalcoholic fatty liver disease, obesity and metabolic syndrome have been extensively attempted in numerous clinical trials [1,2]. Recently, interest in RES has gradually extended to its dietary derivatives, including isorhapontigenin (*trans*-3,5,4′-trihydroxy-3′-methoxystilbene, ISO), oxyresveratrol (*trans*-3,5,2′,4′-tetrahydroxystilbene, OXY) and pterostilbene (*trans*-3,5-dimethoxy-4′-hydroxystilbene, PTS) (Figure 1). Similar to RES, the pharmacological properties of these derivatives such as anti-aging, anti-cancer, anti-diabetic, anti-inflammation, anti-obesity, anti-oxidation, cardio-, hepato-, nephro- and neuro-protection have been observed in various pre-clinical studies [3,4,5,6,7,8]. Notably, the disease-modifying effects of PTS in Alzheimer’s disease, cancer, diabetes and inflammation were commonly superior to RES, while ISO (but not RES) displayed therapeutic potential in chronic obstructive pulmonary disease, an incurable disease affecting hundreds of millions of patients worldwide [4,6,9,10]. Moreover, the oral pharmacokinetic profiles of PTS and ISO were much more favorable than RES [4,6,10,11]. Clearly, it is of great scientific interests to further explore their applications as nutraceuticals.

Metabolomics is a system biology approach involving the comprehensive characterization of metabolites and metabolism in biological systems [12,13]. Unlike genes and proteins, the functions of which are further regulated by epigenetic and post-translational modification mechanisms, respectively, the small molecule metabolites are transformed during metabolism and therefore serve as direct signatures of biochemical processes [12,13]. As metabolomics integrates changes in gene expression, protein levels, enzymatic activity and post-translational modifications, it has advantages over other “omics” approaches. It has been successfully applied in disease diagnosis, the elucidation of the disease/toxicity mechanism, the identification of a novel drug target, the customization of drug treatments and therapeutic outcome monitoring [12,13,14]. Moreover, it has also been increasingly incorporated into nutritional science and food research during the past 10–15 years [15]. Besides the determination of food intake and the classification of individuals into dietary patterns [16], metabolomic profiling also helps elucidate the health-promoting mechanisms of active dietary substances.

To further evaluate the nutraceutical potentials of ISO, OXY and PTS, we assessed their impacts on metabolism and health in Sprague Dawley rats after a 2-week daily oral administration of ISO, OXY or PTS at a dose of 100 µmol/kg/day, using RES as a side-by-side comparator. To capture an integrated snapshot of the entire physiology, metabolomic profiles were assessed in heart, liver, brain tissue homogenates and plasma by gas chromatography–tandem mass spectrometry (GC–MS/MS) through a non-targeted approach. Findings from this pilot nutraceutical study will shed light on the health-promoting mechanisms of these dietary RES derivatives and facilitate their development as functional food supplements.

## 2. Results

### 2.1. Body Weight Gain, Fasting Blood Glucose and Plasma Cholesterol

**Figure 2:**
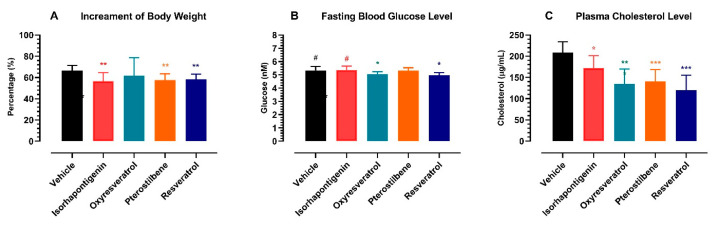
Effects of resveratrol and its dietary derivatives on body wight, fasting glucose and total cholesterol. On day 0, 40 healthy male Sprague Dawley rats were randomly divided in 5 groups (each group: *n* = 8) while their body weight and fasting blood glucose level were measured; during days 1–14, the rats received daily oral administration of RES, ISO, OXY or PTS at the dose of 100 µmol/kg; their body weight and fasting blood glucose level were recorded on day 15 before sacrifice. (**A**) Increment in body weight; (**B**) Fasting blood glucose level; (**C**) Plasma cholesterol level. The blood glucose level was examined with a blood glucose meter while the total cholesterol concentration was monitored with a commercial kit. * *p* < 0.05, ** *p* < 0.01, *** *p* < 0.001 between this group and the vehicle group on day 15 (two-detail unpaired *t*-test); ^#^ *p* < 0.05 between day 0 and day 15 in the same animal (two-detail paired *t*-test).

The effects of RES and its dietary derivatives on body weight increment, fasting blood glucose and plasma cholesterol were assessed in healthy male Sprague Dawley rats after two weeks of daily oral administration of these dietary stilbenes at 100 µmol/kg. When the animals were recruited into the study, their body weights were quite homogenous (178 ± 14 g) and the difference among groups was statistically insignificant (one-way ANOVA: *p* = 0.8533). During the study period, all rats grew rapidly and gained more than 50% in body weight (Figure 2A), suggesting an excellent safety profile for these dietary stilbenes. Similar to RES, which is well known for its protective effects against obesity [17], ISO and PTS also displayed anti-obesity potential as the body increment in rats receiving ISO or PTS was lower than the vehicle group (two-tailed unpaired *t*-test: *p* < 0.01).

The baseline level of fasting blood glucose (FBG) was similar (4.8 ± 0.5 mM) and there was no significant difference among the experimental groups (one-way ANOVA: *p* = 0.5612). In humans, overweight and obesity were associated with diabetes [18]. This association appears to be applicable in rats as well. With a 66.7 ± 4.7% increment in body weight, the fasting blood glucose level in the vehicle group was increased from 4.7 ± 0.2 mM to 5.3 ± 0.3 mM (day 15 versus day 1, two-tailed paired *t*-test: *p* < 0.01). Interestingly, although the rats that received RES, OXY or PTS also gained more than 50% in body weight, their fasting blood glucose levels showed no significant increase (day 15 versus day 1, two-tailed paired *t*-test: *p* > 0.05). On day 15, the fasting blood glucose levels in rats that received RES or OXY were significantly lower than those in the vehicle group (Figure 2B) (two-tailed paired *t*-test: *p* < 0.05).

Similar to RES, which is well known for its cholesterol-lowering activity [17], ISO, OXY and PTS all exhibited such effects as the plasma total cholesterol levels in rats received these dietary stilbenes were significantly lower than in the vehicle group (Figure 2C) (two-tailed paired *t*-test: *p* at least < 0.05). Clearly, besides RES, ISO, OXY and PTS may also possess various health-promoting activities in medical conditions such as obesity, type 2 diabetes and/or cardiovascular conditions.

### 2.2. Metabolomic Analyses

Although the in vivo metabolomics of RES has been reported in various previous studies, the metabolome was mainly profiled in plasma/serum or urine while the metabolomic data in solid organs are sparse [14,19,20,21]. As RES is well known for its health-promoting effects in metabolic syndrome, cardiovascular disease and neuro-degenerative disorder, the metabolomic alternations after two-week stilbene intervention were examined in the tissue homogenates of three specific organs, namely liver, heart and brain. Similarly, the metabolomics was also profiled in plasma samples and applied as an indicator of the impact of the tested stilbenes on general health.

**Figure 3:**
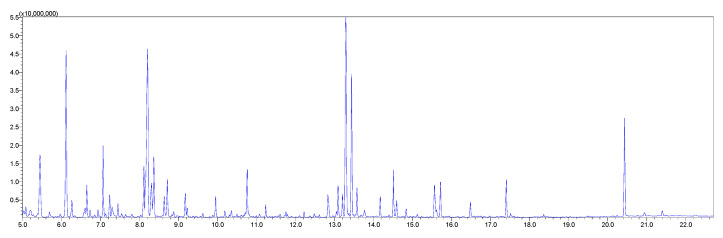
A typical GC-MS/MS total ion chromatogram. This chromatogram was obtained from the plasma sample of a rat dosed with ISO using the Shimadzu Smart Metabolites Database.

The biological samples were processed, derivatized and analyzed by GC-MS/MS. The peak areas of individual metabolites in the Shimadzu Smart Metabolites Database were normalized by the peak area of the internal standard (IS). A typical total ion chromatogram is shown in Figure 3.

**Figure 4:**
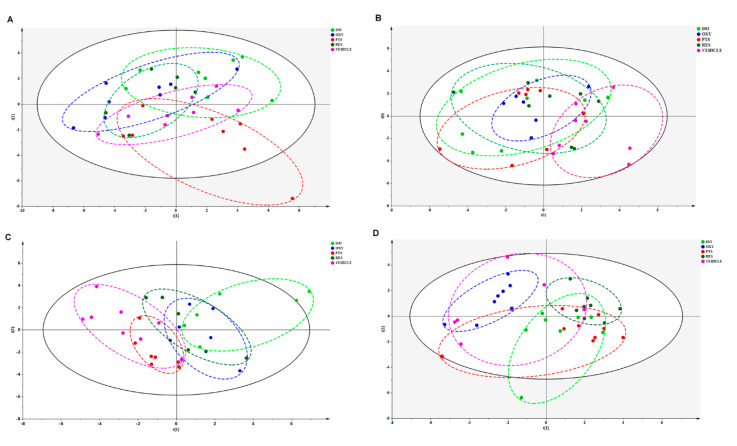
Combined principal component analysis (PCA) scores plot of RES (dark green), OXY (blue), PTS (carmine red), ISO (lime) and vehicle (light deep pink) groups in (**A**) liver, (**B**) heart, (**C**) brain or (**D**) plasma.

The metabolomic shifts after stilbene intervention were analyzed using the partial least square discriminant analysis (PLS-DA) model. As shown in Figure 4, all tested stilbenes displayed distinctly different patterns in the PLS-DA score plots across all tested biological matrices, suggesting different biological activities for each individual stilbene.

#### 2.2.1. Hepatic Metabolomic Analyses

**Figure 5:**
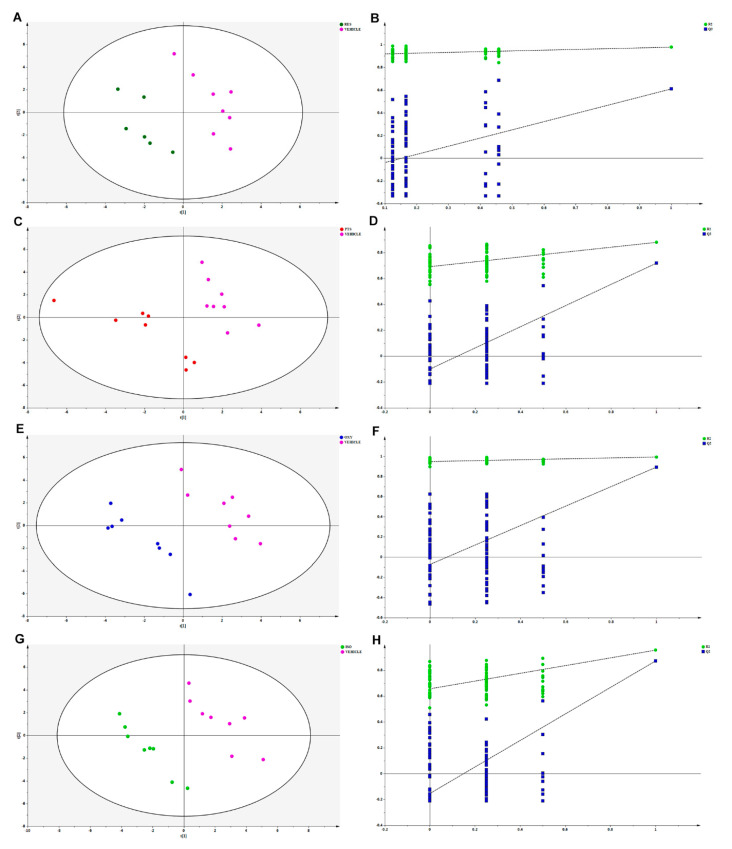
PLS-DA scores plot comparing (**A**) RES, (**C**) PTS, (**E**) OXY and (**G**) ISO with vehicle in liver. Permutation graphs for PLS-DA model in hepatic samples: (**B**) RES, (**D**) PTS, (**F**) OXY and (**H**) ISO.

**Figure 6:**
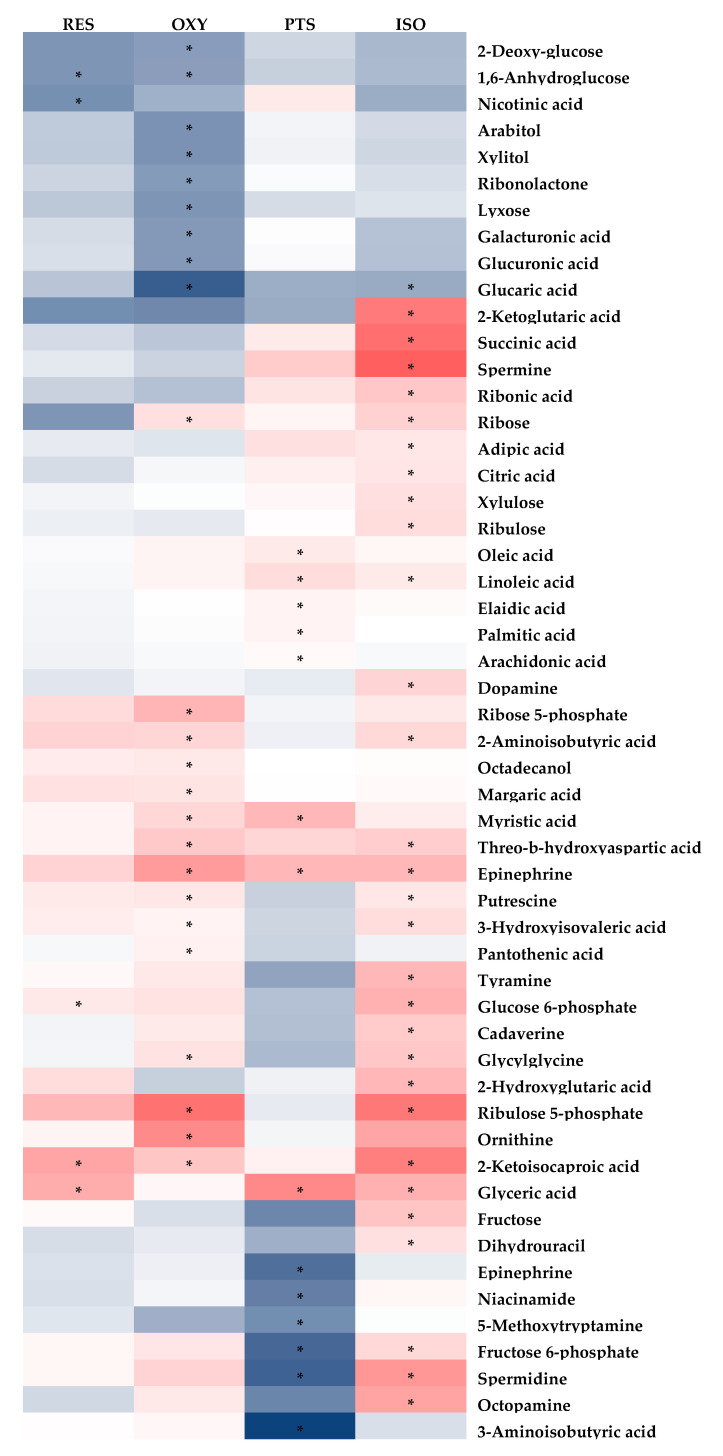

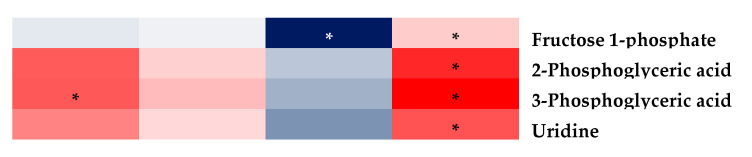
Fold-change heatmap of hepatic metabolites after subjecting fold changes to log 2 transformation and hierarchical clustering via Euclidean distance (blue, white and red colors represent negative, no and positive fold-change, respectively; the darkness of the color represents the degree of fold change). * Indicates that changes in endogenous metabolites are statistically significant (VIP > 1 and FDR < 0.05).

**Figure 7:**
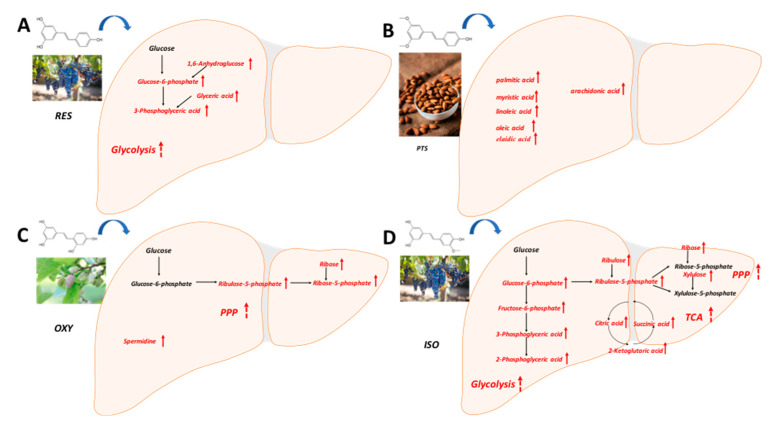
Metabolic pathway analysis in hepatic samples: (**A**) RES, (**B**) PTS, (**C**) OXY and (**D**) ISO.

The PLS-DA model effectively differentiated the rats in any stilbene group from those in the vehicle group (Figure 4A), indicating a shift in metabolomic profiles due to stilbene intervention. The PLS-DA model was validated via 100 permutations, with all Q^2^ regression lines having negative y intercepts (Figure 5) [11,22]. Two liver samples from RES-treated rats were excluded from the analysis due to uninterpretable peaks. A heatmap showing hepatic metabolite changes after stilbene intervention is displayed in Figure 6. The details of metabolite alterations are listed in Supplementary Table S1. The levels of 6, 14, 24 and 34 metabolites changed after two weeks of daily dosing of RES, PTS, OXY and ISO, respectively. Metabolic pathway analysis further showed that RES altered glycolysis; OXY affected the pentose phosphate pathway (PPP) and the biosynthesis of glutathione and spermidine; PTS influenced fatty acid metabolism; while ISO regulated the PPP, tricarboxylic acid (TCA) cycle, glycolysis and glutathione production in hepatic tissue (Figure 7).

#### 2.2.2. Cardiac Metabolomic Analyses

**Figure 8:**
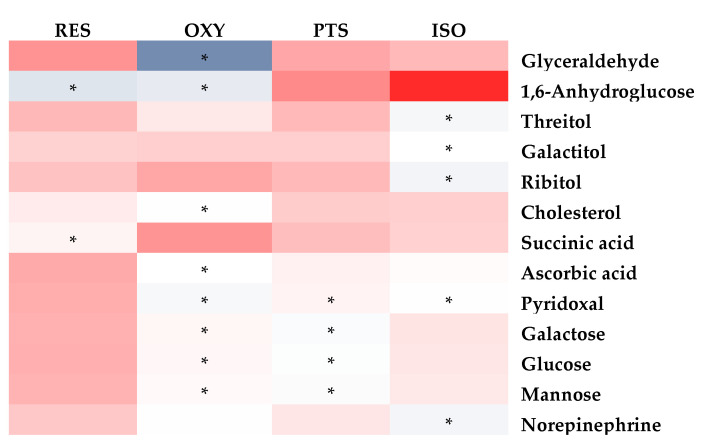

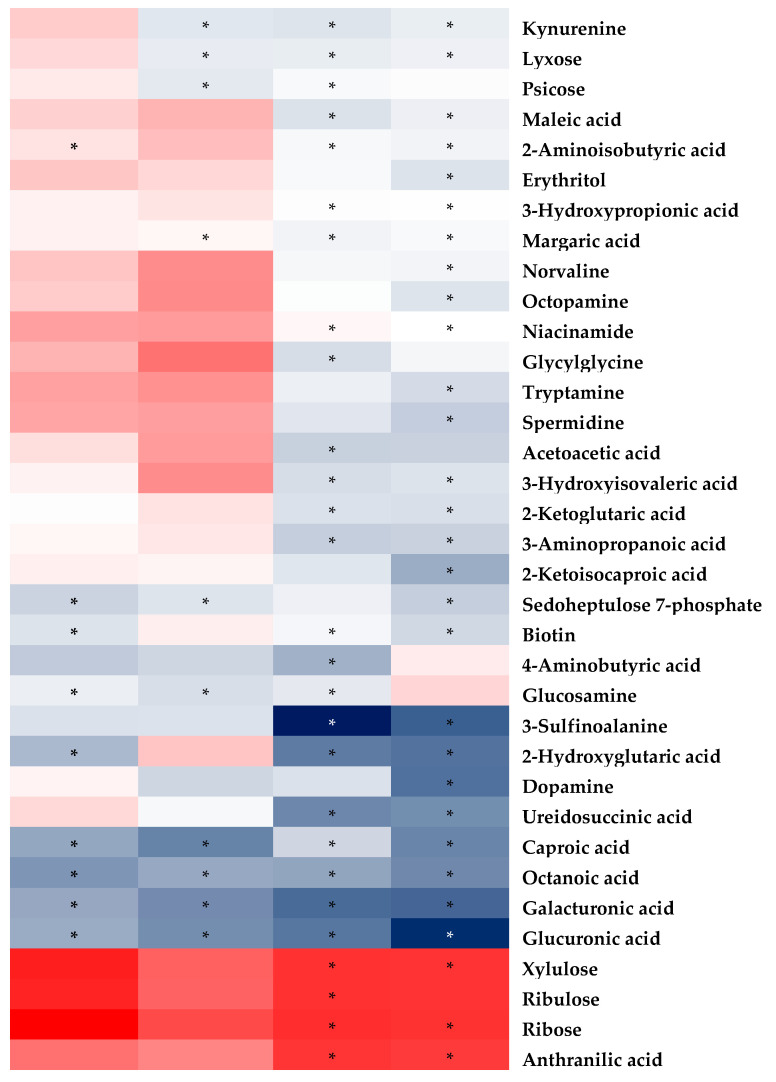
Fold-change heatmap of cardiac metabolites after subjecting fold changes to log 2 transformation and hierarchical clustering via Euclidean distance (blue, white and red colors represent negative, no and positive fold-change, respectively; the darkness of the color represents the degree of fold change). * Altered endogenous metabolites that were statistically significant (VIP > 1 and FDR < 0.05).

**Figure 9:**
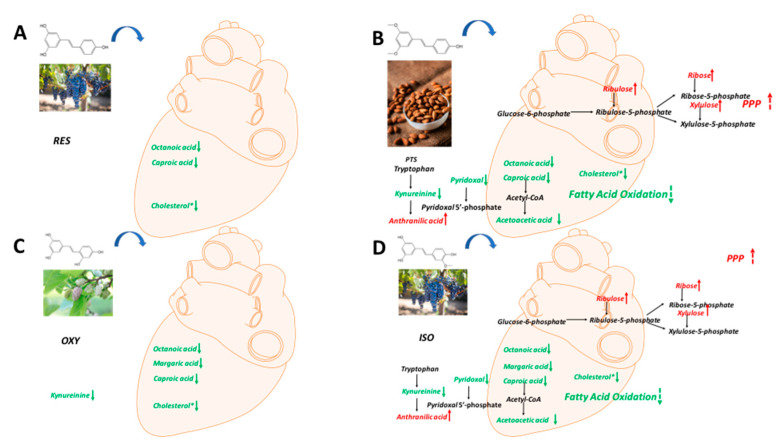
Metabolic pathway analysis in cardiac samples: (**A**) RES, (**B**) PTS, (**C**) OXY and (**D**) ISO.

Similarly, the PLS-DA model differentiated the rats in any stilbene group from those in the vehicle group (Figure 4B), indicating a shift in metabolomic profiles due to stilbene intervention. The PLS-DA model was validated via 100 permutations, with all Q2 regression lines having negative y intercepts (Supplementary Figure S1). A heatmap showing cardiac metabolite changes after stilbene intervention is displayed in Figure 8. The details of metabolite alterations are listed in Supplementary Table S2. The levels of 11, 30, 18 and 35 metabolites changed after two weeks of daily dosing of RES, PTS, OXY and ISO, respectively. Metabolic pathway analysis further revealed that PTS influenced PPP, fatty acids oxidation and tryptophan metabolism while ISO regulated PPP, fatty acids oxidation and tryptophan metabolism in cardiac tissue (Figure 9).

#### 2.2.3. Brain Metabolomic Analyses

**Figure 10:**
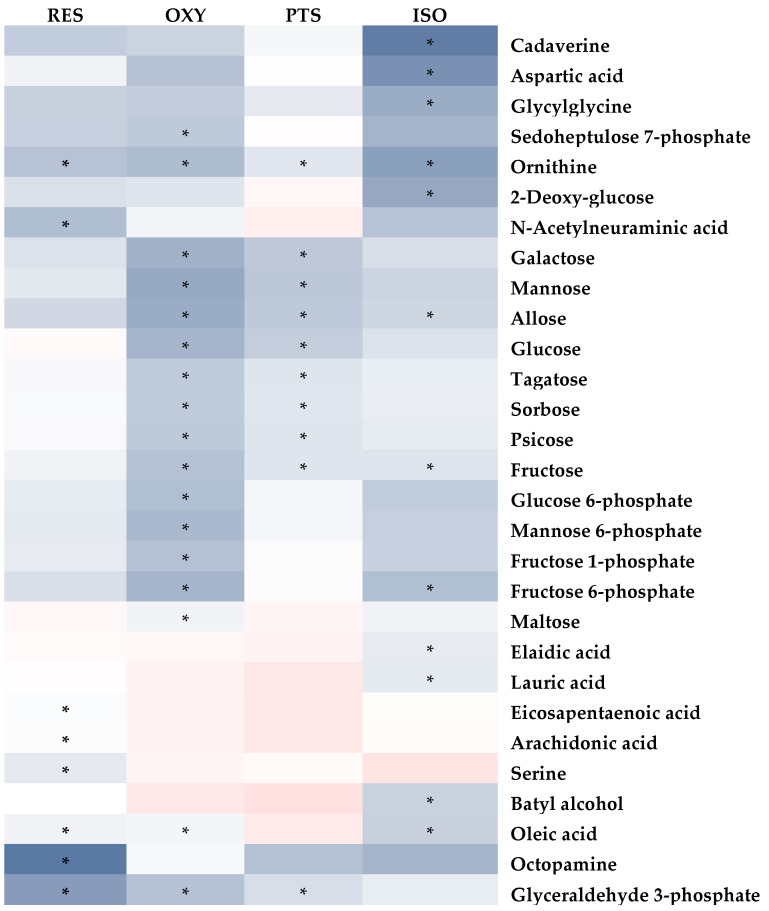

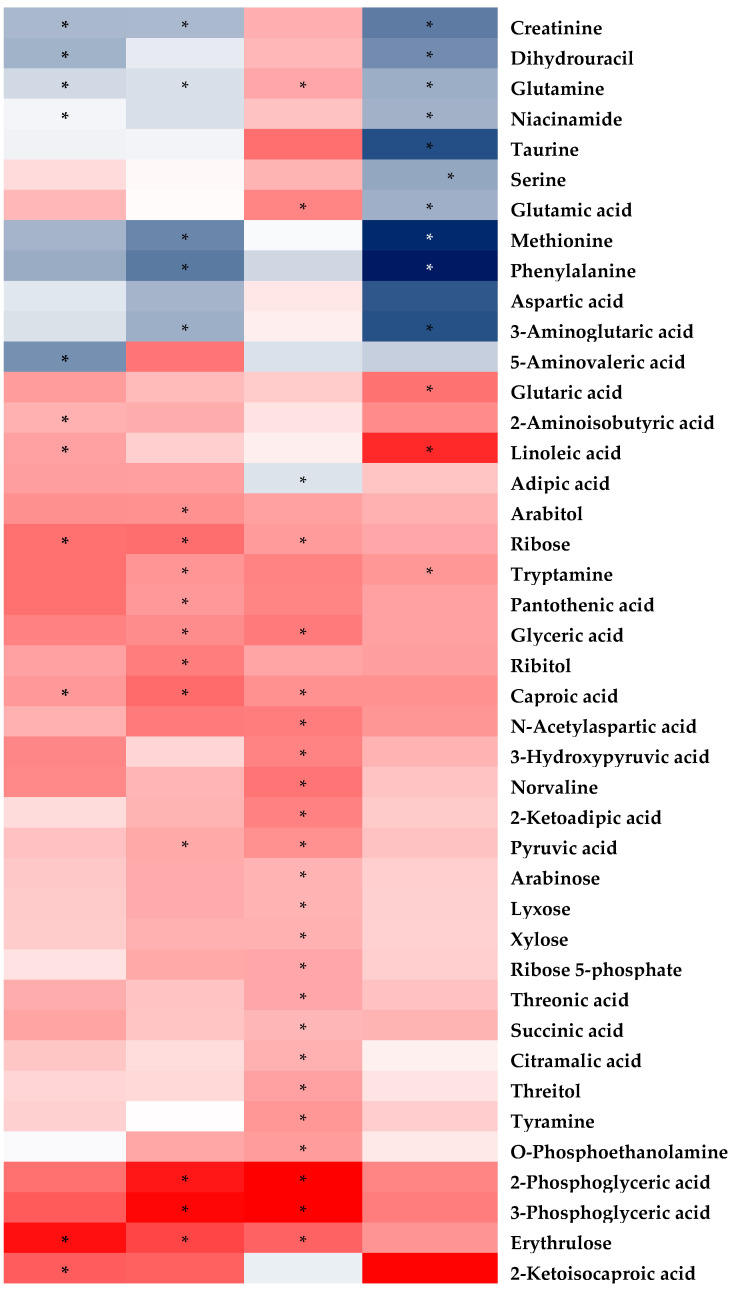
Fold-change heatmap of brain metabolites after subjecting fold changes to log 2 transformation and hierarchical clustering via Euclidean distance (blue, white and red colors represent negative, no and positive fold change, respectively; the darkness of the color represents the degree of fold change). * Altered endogenous metabolites that were statistically significant (VIP > 1 and FDR < 0.05).

**Figure 11:**
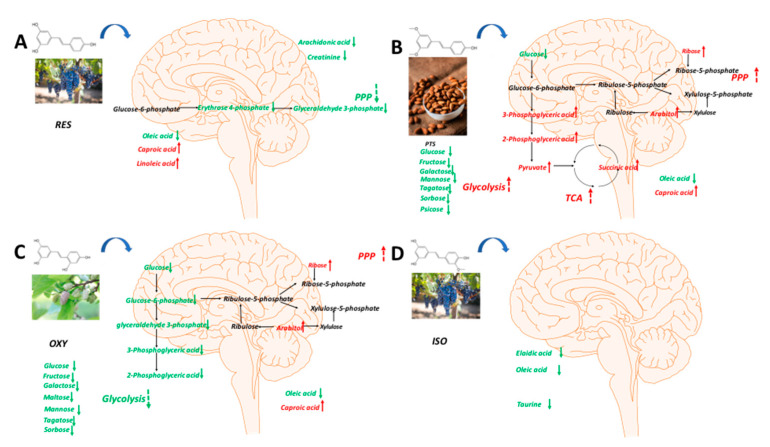
Metabolic pathway analysis in brain samples: (**A**) RES, (**B**) PTS, (**C**) OXY and (**D**) ISO.

Again, the PLS-DA model differentiated the rats in any stilbene group from those in the vehicle group (Figure 4C), demonstrating a shift in metabolomic profiles due to stilbene intervention. The PLS-DA model was validated via 100 permutations, with all Q^2^ regression lines having negative y intercepts (Supplementary Figure S2). The details of metabolite alterations are listed in Supplementary Table S3. A heatmap showing brain metabolite changes after stilbene intervention is displayed in Figure 10. The levels of 21, 34, 35 and 26 metabolites changed after two weeks of daily dosing of RES, PTS, OXY and ISO, respectively. Metabolic pathway analysis further indicated that RES altered PPP; OXY influenced PPP and glycolysis; PTS regulated PPP, fatty acids oxidation and glycolysis (Figure 11).

#### 2.2.4. Plasma Metabolomic Analyses

**Figure 12:**
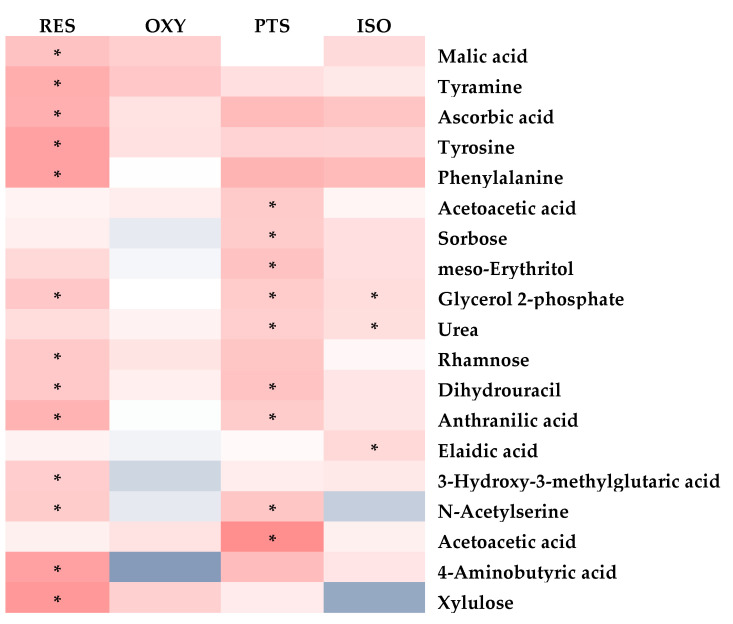

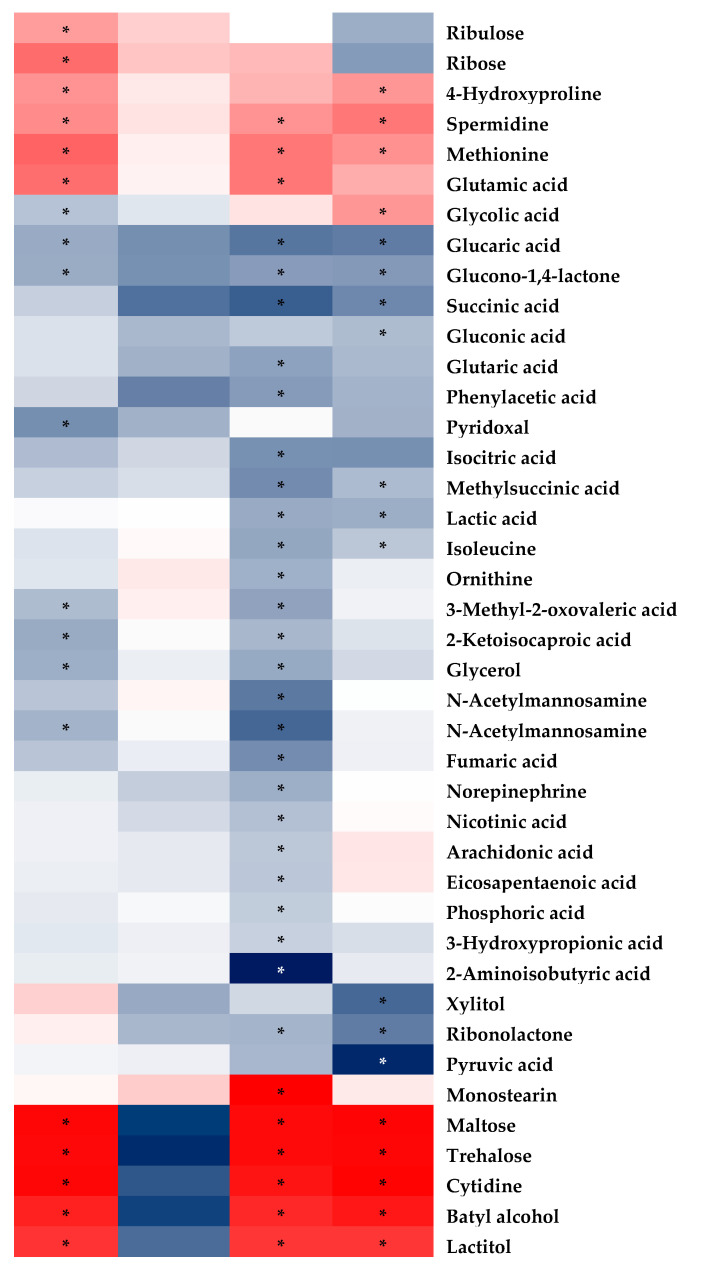
Fold-change heatmap of plasma metabolites after subjecting fold changes to log 2 transformation and hierarchical clustering via Euclidean distance (blue, white and red colors represent negative, no and positive fold change, respectively; the darkness of the color represents the degree of fold change). * Altered endogenous metabolites that were statistically significant (VIP > 1 and FDR < 0.05).

**Figure 13:**
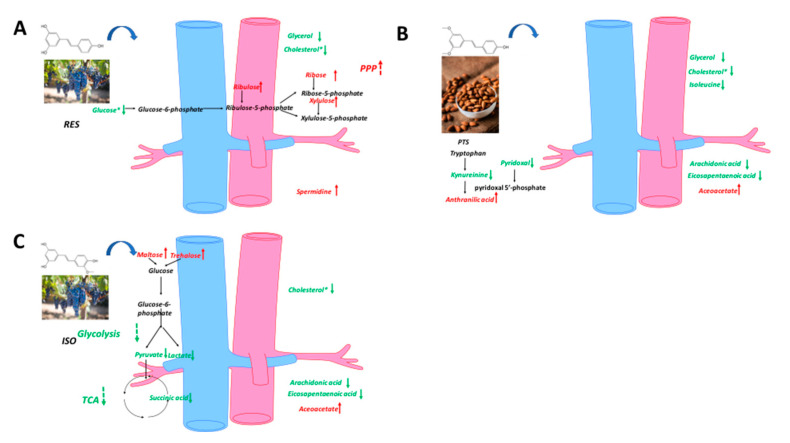
Metabolic pathway analysis in plasma samples: (**A**) RES, (**B**) PTS, (**C**) ISO.

Unsupervised PCA clustering in plasma samples demonstrated a distinction between RES, PTS and ISO-treated groups and the vehicle group, as indicated by their incomplete overlapping ellipses (Figure 4D). However, what was almost an overlap the OXY-treated group and the vehicle indicated an absence of inter-group separation (Figure 4D). PLS-DA model validation was performed via 100 permutations to assess data overfitting for the plasma samples, and the Q^2^ regression line for all permutation graphs had negative y intercepts, thus validating the PLS-DA models (Supplementary Figure S3). The details of metabolite alterations are listed in Supplementary Table S3. A heatmap showing plasma metabolite changes after stilbene intervention is displayed in Figure 11. The levels of 32, 39 and 22 metabolites changed after two weeks of daily dosing of RES, PTS and ISO, respectively (Figure 12). Notably, there is an absence of a significant change in metabolites in rat plasma following the administration of OXY. Pathway analysis revealed that RES modulated PPP; PTS influenced the spermidine biosynthesis and tryptophan metabolism; while ISO regulated the TCA cycle and glycolysis (Figure 13).

## 3. Discussion

In this study, the nutraceutical potentials of ISO, OXY and PTS were assessed in Sprague Dawley rats after a two-week oral administration (100 µmol/kg/day), using RES as a comparator. Significant in vivo health benefits of these stilbenes were observed, as the rats that received ISO, PTS or RES showed reduced body weight gain; the rats that received OXY or RES displayed improved FBG levels; while all of the tested stilbenes exhibited cholesterol-lowering effects. Notably, the anti-obesity effects of ISO and PTS, the anti-diabetic potential of OXY and the cardio-protective activities of ISO, OXY and PTS were identified. To further elucidate their mechanisms of action, metabolomic profiling was conducted using GC-MS/MS on hepatic, cardiac, brain tissues and plasma samples.

### 3.1. Hepatic Metabolomics

A lower level of 1,6-anhydroglucose, a source of glucose-6-phosphate [23], and higher levels of glucose-6-phosphate, 3-phosphoglyceric acid and glyceric acid, which are intermediates of glycolysis, were observed in the liver after RES treatment. These findings indicate enhanced glycolysis in the liver, which may explain the decreased blood glucose levels following RES intervention.

The anti-obesity benefit of PTS could be explained by increased lipolysis, followed by increased free fatty acid mobilization to the liver [24], as evidenced by the higher hepatic levels of palmitic acid, myristic acid, linoleic acid, arachidonic acid, oleic acid and elaidic acid. This hypothesis is further supported by increased hepatic epinephrine levels, as catecholamines are potent catabolic promoters and lipolysis stimulants [25]. Additionally, the increased level of acetoacetate in plasma, a product of fatty acid oxidation, indicates enhanced lipolysis in the liver, as acetoacetate can serve as a circulating energy source from the liver to other organs.

PPP is upregulated by OXY, as indicated by the increased levels of ribulose-5-phosphate, ribose and ribose-5-phosphate, which are intermediates of the non-oxidative phase of PPP. Besides being an essential pathway for nucleotide synthesis [26], PPP generates NADPH for the maintenance of the reduced state of endogenous antioxidants, which, in turn, scavenge reactive oxygen species and free radicals [27]. Excessive hepatic oxidative stress contributes to the initiation and progression of liver injury, and severe liver diseases like non-alcoholic steatohepatitis [28]; hence, the upregulation of PPP by OXY may be liver-protective. The antioxidant benefits of OXY in the liver are further corroborated by increased levels of pantothenic acid, which boosts the biosynthesis of glutathione, a major endogenous antioxidant, by enhancing cell energetics [29]. The increased hepatic production of spermidine, a naturally occurring polyamine, was inferred from the elevated levels of its precursors, ornithine and putrescine [30]. Spermidine in the liver has been demonstrated to prevent liver fibrosis and hepatocellular carcinoma by relieving cancer-related defects in autophagy, thereby ameliorating oxidative stress-induced cell death [31]. In summary, OXY supplementation may yield liver protective benefits, primarily through the upregulation of liver antioxidants, which is consistent with an in vivo study on rats with ethanol-induced hepatic dysfunction [32]. Although a similar lipolysis pattern was observed with OXY (elevated levels of epinephrine and fatty acids), no significant body weight loss was observed after OXY treatment.

After ISO dosing, enhanced glycolysis was evidenced by higher hepatic levels of 2-phosphoglyceric acid, 3-phosphoglyceric acid, glucose 6-phosphate and fructose 6-phosphate; upregulated PPP was suggested by elevated levels of glucose-6-phosphate, ribulose-5-phosphate, ribulose, xylulose and ribose; and increased TCA activity was demonstrated by augmented levels of citric acid, succinic acid and 2-ketoglutaric acid. As discussed previously, the upregulation of PPP confers higher antioxidant properties in the liver via the increased generation of NADPH, ultimately contributing to hepato-protective benefits against oxidative stress [26]. The in vivo antioxidant property of ISO was reinforced by an increase in the production of the most abundant endogenous antioxidant, glutathione, as suggested by raised levels of putrescine, cadaverine, spermidine and spermine, which are intermediates in the complex cascade of glutathione metabolism, as indicated in the Kyoto Encyclopedia of Genes and Genomes (KEGG). Increased TCA activity indicated that ISO might exert a hepato-protective effect via the activation of mitochondrial function, as mitochondrial dysfunction is related to the majority of chronic liver diseases [33]. Enhanced glycolysis also suggested that ISO could exert an anti-diabetic effect. Although ISO did not alter fasting blood glucose in this study, its anti-diabetic potential has been reported in mice [34]. Similarly to OXY, the lipolysis effect of ISO might be induced by increased levels of epinephrine and fatty acids.

In summary, a hepatic metabolomic examination showed that ISO might exert a hepato-protective effect via the upregulation of PPP, which relieves oxidative stress, and TCA, which activates mitochondrial function. OXY might exhibit a liver-protective effect by upregulating PPP. RES can stimulate glycolysis, which is consistent with the decreased fasting blood glucose, while no significant metabolite was discovered related to liver protection. The body weight decrement in PTS and ISO may be explained by enhanced lipolysis in the liver.

### 3.2. Cardiac Metabolomics

A global decrease in the levels of identified metabolites was observed in cardiac tissues after the intake of RES, PTS, OXY or ISO, except for metabolites related to the PPP, which were increased in the PTS and ISO groups. Additionally, GC-MS/MS analysis confirmed that total cholesterol levels decreased in all the treatment groups, reaffirming the cardioprotective potentials of RES, PTS, OXY and ISO [35].

In cardiac tissue, fatty acids and glucose are the two major energy sources. Higher fatty acid metabolism produces more reactive oxygen species (ROS) and oxygen waste, as it requires more oxygen than glucose metabolism [36,37]. The levels of fatty acids, octanoic acid and caproic acid were decreased after RES treatment, indicating reduced fatty acid use in the heart, which is beneficial to the heart [37]. Additionally, the heart protection effect was reinforced by the lower plasma cholesterol level after RES intervention, as hypercholesterolemia is a risk factor associated with coronary heart disease [35]. This finding is consistent with previous studies showing that RES can exert cardioprotective effects [38].

PTS also exhibited the ability to lower fatty acid levels in the heart, including octanoic acid and caproic acid. Additionally, acetoacetate, a ketone, displayed a lower level in the heart after PTS treatment, indicating the downregulation of fatty acid oxidation. Thus, PTS may confer a cardioprotective effect via the downregulation of the fatty acid metabolism [37]. The upregulation of the PPP pathway, whose product can inhibit oxidative stress [27], was observed as the ribose, xylulose and ribulose levels increased after PTS intervention. Collectively, PTS may exert a cardioprotective effect by suppressing oxidative stress in the heart because ROS plays a significant role in heart failure [39]. This is similar to a previous finding showing that PTS can protect against heart ischemia-reperfusion injury by suppressing oxidative stress [40]. The tryptophan metabolism was altered after PTS intervention. PTS may increase the level of pyridoxal 5′-phosphate, whose decreased level is associated with cardiovascular disease, as the increased level of anthranilic acid, which is positively correlated with pyridoxal 5′-phosphate, was observed [41]. Thus, the PTS intervention may exert a cardioprotective effect by improving the pyridoxal 5′-phosphate status. This finding is reinforced by the decreased level of pyridoxal, a precursor of pyridoxal 5′-phosphate [42].

Treatment with OXY decreased the levels of fatty acids (octanoic acid, margaric acid and caproic acid) and cholesterol in the heart, indicating reduced fatty acid oxidation. Combined with the decreased plasma cholesterol level, OXY intervention may exert a cardioprotective effect.

ISO intervention caused an upregulation of PPP, manifested as increased levels of ribulose, ribose and xylulose, and a reduction in fatty acid metabolism, as indicated by decreased levels of octanoic acid, margaric acid, caproic acid and acetoacetic acid in the heart. Both findings suggest that ISO can exert a cardioprotective effect by suppressing oxidative stress. Similar to PTS, ISO may confer cardioprotective benefits through the modulation of tryptophan metabolism [41], as evidenced by increased anthranilic acid and decreased pyridoxal levels observed after ISO treatment.

Collectively, RES and OXY can exert a cardioprotective effect by reducing fatty acid metabolism in the heart, while ISO and PTS can display cardioprotective effects by reducing fatty acid metabolism, upregulating PPP in the heart, and modulating tryptophan metabolism.

### 3.3. Brain Metabolomics

Identified metabolites were observed to generally decrease in the brain after treatment with RES, PTS, OXY or ISO.

Arachidonic acid, a precursor of inflammatory messengers [43], was decreased in the brain after RES treatment, indicating that RES may exert a neuroprotective effect via anti-inflammation. Creatinine, whose concentration in the brain is positively correlated with brain aging [44], was observed at lower levels following RES intervention, suggesting that RES may protect the brain from aging. RES treatment decreased long-chain fatty acid oleic acid, while increasing medium-chain fatty acid caproic acid, which can facilitate neurite outgrowth [45]. Notably, linoleic acid, an essential fatty acid crucial for the central nervous system [46], was increased in the brain after RES intervention. However, the PPP pathway, which produces NADPH for the reduction in oxidative stress [27], was inhibited (decreased erythrose 4-phosphate and glyceraldehyde 3-phosphate), indicating that RES may not reduce oxidative stress through the PPP pathway.

Similarly to RES, medium-chain fatty acid caproic acid was increased, while long-chain fatty acid oleic acid was decreased after OXY intervention, which is beneficial to the brain [45]. The PPP pathway was upregulated, as indicated by increased levels of ribose and arabitol, suggesting that OXY may protect the brain via the inhibition of oxidative stress response [47]. This is consistent with a previous study showing that OXY can exert anti-Parkinsonian effects by suppressing oxidative stress [48]. OXY intervention also triggered a global decrease in sugar levels, including glucose, sorbose, psicose, tagatose, fructose, mannose, galactose, maltose and allose and an inhibition of glycolysis, as indicated by the reduction in glycerol 2-phosphate, glucose 6-phosphate, 2-phosphoglyceric acid, 3-phosphoglyceric acid and glyceraldehyde 3-phosphate, suggesting that brain sugar uptake was inhibited by OXY. The decreased sugar levels, especially glucose, indicate that OXY may be beneficial for Alzheimer’s disease patients, as higher brain glucose levels are correlated with Alzheimer’s disease [49]. This is supported by a previous finding showing that OXY may treat Alzheimer’s disease via the inhibition of β-secretase [50]. Additionally, pyruvate, which exerts neuroprotective effects via the activation of the hypoxia-inducible factor-1α and erythropoietin signaling cascade [51], was increased in the brain after OXY intervention, indicating that OXY may also display neuroprotection through a similar pathway.

In the brain, several metabolic pathways were upregulated after PTS treatment, including the PPP (higher levels of ribose and ribose 5-phosphate), which can produce NADPH to scavenge ROS [27]; glycolysis (increased 3-phosphoglyceric acid, 2-phosphoglyceric acid, glyceric acid and pyruvic acid), whose increased level is associated with neuroprotection [52]; and the TCA cycle (elevated succinic acid and pyruvic acid), whose suppression is a common pathological mechanism in neurodegeneration [53]. These results indicate that PTS may exert neuroprotective effects via the suppression of oxidative stress, the upregulation of glycolysis and the activation of mitochondrial function. Notably, sugar levels were significantly decreased in the brain while glycolysis was enhanced, indicating that the lower glucose level is caused by glycolysis enhancement rather than glucose uptake inhibition. This is a more favorable pattern [49] than that observed with OXY, whose glycolysis is inhibited.

Although a decreased level of long-chain fatty acids such as elaidic acid and oleic acid was observed, ISO intervention did not exhibit a favorable neuroprotective metabolic pattern: there was no upregulation of the PPP, no activation of the TCA cycle and a seemingly decreased glycolysis, as indicated by the declined level of fructose 6-phosphate, with no reduction in glucose levels. Additionally, taurine, a neuromodulator that can protect the brain against neuronal overexcitation and glutamate-induced neurotoxicity [54], was decreased after ISO treatment.

Conclusively, PTS appears to have the highest potential for neuroprotective effects due to its multiple favorable metabolic pathway changes. OXY exhibits several beneficial metabolic changes in the brain, including decreased glucose and increased pyruvate levels, and an upregulation of PPP. However, RES and ISO do not show significant metabolic changes beneficial to the brain. Notably, no study has demonstrated the neuroprotective effect of ISO. While RES has been reported to exhibit neuroprotective abilities [55,56], a study has shown that PTS, but not RES, is more effective in modulating aging and Alzheimer’s disease [57]. Thus, PTS and OXY may have greater potential for neuroprotective effects.

### 3.4. Plasma Metabolomics

The FBG of RES-treated rats was significantly lower than that of the vehicle group despite the freely available food source, suggesting that RES may have anti-diabetic effects, consistent with the findings from a recent study [58]. The mechanism underlying the sugar-regulating properties of RES could be explained by the decreased levels of 2-ketoisocaproic acid in plasma. High amounts of circulating 2-ketoisocaproic acid inhibit insulin-stimulated glucose transport in an mTORC-1-dependent manner, which contributes to insulin resistance and ultimately results in type 2 diabetes mellitus [59]. It has also been demonstrated that RES directly inhibits mTORC-1 phosphorylation [60], thereby decreasing mTORC-1 activity and reducing the risk of diabetes associated with hyperactive mTOR [61].

The weight management benefits of RES were also confirmed, as the percentage weight increment in RES-treated rats was significantly lower than that of the vehicle group. This observation is supported by the lower plasma glycerol levels detected in the RES-treated group. Glycerol, along with free fatty acids, is a product of the intracellular lipolysis of triglycerides [62]. Contrary to the conclusion of a systematic review stating that the circulating levels of free fatty acids and glycerol are notably elevated in obesity [62], the lower levels of plasma glycerol coupled with reduced weight gain in RES-treated rats demonstrate anti-obesity benefits.

The weight management benefits of RES were also elucidated, as the percentage weight increment in RES-treated rats was significantly lower than that of the vehicle group. This observation is supported by the lower plasma glycerol levels detected in the RES-treated group. Glycerol, along with free fatty acids, is a product of the intracellular lipolysis of triglycerides [62]. In contrast to the conclusion of a systematic review stating that the circulating levels of free fatty acids and glycerol are notably elevated in obesity [62], the lower levels of plasma glycerol coupled with reduced weight gain in RES-treated rats demonstrate anti-obesity benefits.

A marked increase in PPP intermediates in plasma, namely ribulose, ribose and xylulose, suggests the activation of the PPP in response to RES. The PPP performs numerous biological functions, including the generation of nicotinamide adenine dinucleotide phosphate (NADPH) [26]. Since NADPH is a major source of reductant in non-photosynthetic cells [26], the activation of the PPP by RES provides enhanced protection against oxidative stress.

Plasma spermidine was elevated following RES intervention. Spermidine demonstrates anti-inflammatory properties, preserves mitochondrial function, and prevents stem cell senescence, which collectively contribute to its anti-aging benefits [63]. In vivo studies have also shown that spermidine enhances cardiovascular health by preserving diastolic function and reducing cardiac hypertrophy [64]. Together with the reduced plasma total cholesterol level after RES intervention, the elderly population with high cardiovascular risk factors may benefit from RES supplementation. The elevated level of spermidine and decreased level of plasma total cholesterol were also observed in PTS and ISO treatments, indicating that they may also confer anti-aging and cardio-protective effects.

Increased plasma anthranilic acid and decreased pyridoxal levels were observed after RES treatment, indicating that RES may display cardioprotective effects via the modulation of the tryptophan metabolism, as previously mentioned [41].

PTS intervention decreased arachidonic acid in the plasma, suggesting anti-inflammatory benefits through the downregulation of systemic inflammatory messengers. Moreover, a recent in vivo study demonstrated the ability of arachidonic acid to aggravate existing obesity and obesity-induced complications by modifying adipocyte browning and altering gut microbiota [65]. Therefore, the downregulation of circulating arachidonic acid not only signifies the anti-inflammatory properties of PTS but also provides evidence supporting its anti-obesity benefits. PTS could be used for weight management, as indicated by the significantly lower weight gain in PTS-treated rats, and as complemented by lower circulating glycerol levels, which are typically high in obese subjects [62] and lower isoleucine levels. Isoleucine, a branched-chain amino acid, is highly associated with obesity in children and adolescents when elevated [62], and serves as a predictor for diabetes as well as a biomarker for future insulin resistance [66]. PTS intervention increased anthranilic acid and decreased pyridoxal levels in plasma, similar to the effects observed in the heart. Together with the decreased plasma cholesterol level, this indicates that PTS has cardio-protective effects. The decreased levels of fatty acids, arachidonic acid and eicosapentaenoic acid in plasma, along with the increased levels of fatty acids in the liver, suggest the mobilization of fatty acids from plasma to the liver, which subsequently triggers enhanced lipolysis and fatty acid oxidation in the liver. This explains the anti-obesity effect of PTS. This is further reinforced by the increased circulating level of acetoacetic acid, a product of excessive acetyl-CoA from fatty acid oxidation.

An intervention with ISO demonstrated weight management benefits, as shown through the significantly lower percentage weight increment in ISO-treated rats relative to the vehicle. This is further supported by a significant decrease in isoleucine, which in high circulating concentrations is correlated with obesity [67] and serves as a biomarker for insulin resistance [66]. The TCA cycle is possibly downregulated, as indicated by the decreased circulating TCA intermediate, succinic acid. This is coupled with downregulation in the preceding process—glycolysis, as indicated by the reduced plasma levels of pyruvic acid and lactic acid, which are the end products of aerobic [68] and anaerobic glycolysis [69], respectively. Additionally, there was a drastic accumulation of two disaccharides, maltose and trehalose, which can be hydrolyzed into their component monosaccharide, glucose [70], before feeding into glycolysis. However, an opposite pattern was observed in the liver; the ISO intervention caused enhanced TCA and glycolysis in the liver. Thus, the decreased circulating levels of intermediates of TCA and glycolysis may result from the increased consumption of these intermediates in the liver. Collectively, ISO intervention enhanced the TCA cycle and glycolysis in the liver, the major metabolic organ, indicating that ISO may activate mitochondrial function.

### 3.5. Structure–Activity Relationship

Despite their structural similarities, the biological activities and metabolic effects of these stilbenes appear to be distinct. While elucidating the structure–activity relationship of stilbenes is of great scientific interest, it is unlikely to be fully achieved using a global/untargeted metabolomic approach, even with a database containing information on 475 small molecule metabolites. Additionally, like many natural products, these stilbenes have multiple molecular targets, which complicates efforts to assess their structure–activity relationships. Compared to PTS, ISO and OXY may share more similarities with RES in terms of biological activity, as both possess a 3,5-dihydroxyl structure. Beyond hydroxyl groups, the presence of methoxy groups also significantly impacts their activities; for example, ISO, but not RES, has shown therapeutic potential in chronic obstructive pulmonary disease [4]. RES is well known for its poor pharmacokinetic properties, characterized by a short half-life, extensive phase II metabolism and low bioavailability. Fortunately, ISO, OXY and PTS exhibit much more favorable pharmacokinetic profiles. The structure–pharmacokinetic relationship of these compounds has been discussed in our previous publications [4,5,6,10,11].

### 3.6. Future Perspectives

Due to the exploratory nature of this study, the impacts of RES and its dietary derivatives on metabolomics were only assessed by GC-MS/MS using a commercial database that contains information on 475 small molecule metabolites. Consequently, alterations in metabolites not included in this database could not be detected. Therefore, alternative analytical technologies, such as LC-MS/MS and/or larger metabolite databases, are advocated for future studies to obtain a more comprehensive picture of the entire physiology. Similarly, as an initial attempt to capture a snapshot of the impacts of RES and its dietary derivatives, only a global/untargeted metabolomic approach was applied. To facilitate clinical translation, future studies on the health-promoting effects of RES and its dietary derivatives should focus on specific diseased conditions. A targeted metabolomic approach appears to be a powerful tool for dissecting the pharmacological mechanisms of these dietary stilbenes. The selection of metabolites in targeted metabolomic studies largely depends on the pathological conditions being examined. It is hoped that the findings from this study will help guide the design of more effective metabolomic investigations in the future.

## 4. Materials and Methods

### 4.1. Special Precautions

Due to the light-sensitive nature of stilbene compounds, all laboratory handling of RES, ISO, PTS and ISO was executed under dimly lit conditions [11].

### 4.2. Chemicals and Reagents

Isorhapontigenin (trans-3,5,4′-trihydroxy-3′-methoxystilbene, ISO), oxyresveratrol (trans-3,5,2′,4′-tetrahydroxystilbene, OXY), pterostilbene (trans-3,5-dimethoxy-4′-hydroxystilbene, PTS) and resveratrol (trans-3,5,4′-trihydroxystilbene, RES) were purchased from Tokyo Chemical Industry (Tokyo, Japan). The internal standard (IS), myristic-d_27_ acid (mass shift: M+27; isotopic purity: 98%) and suspending agent, sodium salt of carboxymethyl cellulose (CMC), were obtained from Sigma-Aldrich (St. Louis, MO, USA). Derivatization reagents, 2% methoxyamine chloride in pyridine and N-Methyl-N-(trimethylsilyl)trifluoroacetamide, were obtained from Thermo Fisher Scientific (Waltham, MA, USA). Methanol, acting as protein precipitant, was supplied by Tedia (Fairfield, OH, USA). Ultra-pure water (18.2 MΩ·cm at 25 °C) was dispensed from a Millipore Direct-Q^®^ ultra-pure water system (Billerica, MA, USA) and used throughout the study.

### 4.3. Animals

This metabolomic study was conducted with strict adherence to the “Guidelines on the Care and Use of Animals for Scientific Purposes” (Singapore), which align with the ARRIVE guidelines [71]. The study design and animal handling procedures were reviewed and approved by the Institutional Animal Care and Use Committee of the National University of Singapore (NUS) (R15-1273). All in vivo experiments were carried out in a centralized specific pathogen-free animal facility (temperature: 22 ± 1 °C; humidity: 60–70%) operated by Comparative Medicine, NUS. Healthy Sprague Dawley rats (male, ~7 weeks old, weight: 178 ± 14 g) were purchased from InVivos (Singapore). The animals were housed under a 12 h light–dark cycle with free access to food and water except that food supply was ceased for at least 12 h before measuring fasting blood glucose (FBG) level.

Forty rats were randomized into 5 groups (each group: *n* = 8). Group 1 received 0.3% (*w*/*v*) CMC (vehicle) at 3.87 mL/kg, while groups 2–5 received daily oral administration of aqueous suspension of RES, ISO, OXY or PTS, respectively, at a dose of 100 µmol/kg (same volume as the vehicle group) in the morning during days 1–14. Besides on day 1 and day 15, the body weight was monitored twice a week during the dosing period to adjust the actual dose of each compound. Fasting blood glucose level was measured on day 1 before first dosing and on day 15 before the animals were sacrificed. The rats were euthanized with carbon dioxide, the plasma and organ samples were harvested and stored at −40 °C before further sample analysis.

### 4.4. Blood Glucose and Plasma Cholesterol

After overnight fasting, a small puncture was made in the tail vein with a 23G needle. Subsequently, a drop of blood was milked and its glucose level was measured with an Accu-Chek Performa blood glucose meter (Roche Diagnostic, Mannheim, Germany). The plasma total cholesterol level was determined by a Cholesterol Quantification Kit (Sigma-Aldrich, St. Louis, MO, USA) according to the manufacturer’s guidance.

### 4.5. Sample Preparation and Metabolomic Profiling by GC-MS/MS Analyses

All biological samples were first thawed to room temperature. Approximately 200 mg of liver, heart or brain tissue were minced finely and put into separate Eppendorf tubes. After addition of ultra-pure water at a ratio of 3 µL/mg, homogenization was carried out by a Bullet Blender Gold homogenizer (Next Advance Inc, Troy, NY, USA).

The biological sample preparation and derivatization procedures were carried out with our established protocol [11,72,73]. Briefly, protein precipitation was conducted by adding 200 µL of myristic-d27 acid methanol solution (internal standard, 2 µg/mL) to 30 µL of plasma or tissue homogenates. After 5 min of rigorous vortex mixing at room temperature, the samples were centrifuged at 15,000× *g* (4 °C) for 10 min. Then, 180 µL of the supernatant was transferred to a glass tube and dried under nitrogen gas. The residues were suspended in 100 µL of toluene, vigorously vortexed for 10 s and dried again under nitrogen. A two-step derivatization was applied to derivatize the metabolites. The samples were first mixed with 50 µL of 2% methoxyamine chloride in pyridine and oximated at 60 °C for 1.5 h. Subsequently, silylation was carried out by adding 50 µL of N-Methyl-N-(trimethylsilyl)trifluoroacetamide and further incubating at 60 °C for 1 h. After derivatization, 80 µL of the samples were transferred to glass vials for GC-MS/MS analysis. Pure water was used as a blank, while pooled samples from the same matrix of different rats were used as quality control (QC). Blank samples were injected at the beginning and end of each sample batch, and QC samples were analyzed between every five individual study samples.

All metabolomic samples were analyzed by a Shimadzu TQ8040 gas chromatography–triple quadrupole mass spectrometer (Shimadzu Corporation, Kyoto, Japan) following an established protocol [11,18]. Chromatographic separation was obtained with a capillary column (BPX-5, 30 m × 0.25 mm × 0.25 µm, SGE Analytical Science, Ringwood, Victoria, Australia) through the constant delivery of the helium carrier gas at the flow rate of 1.14 mL/min. The temperature gradient schedule was (a) 0.0–2.0 min: 60 °C; (b) 2.0–20.0 min: 60 → 330 °C (c) 20.0–23.0: min 330 °C. The interface and ion source were operated at 280 °C and 200 °C, respectively. The injection volume was 1 µL with a split ratio of 1:10 while the injector port was set at 250 °C. This mass spectrometer was operated in multiple reaction monitoring (MRM) mode.

### 4.6. Metabolomic Data Analyses

The data analyses were performed with our established protocol [11,18]. The identification of small molecule metabolites in biological samples was based on the Shimadzu Smart Metabolites Database (Shimadzu Corporation, Kyoto, Japan), which contains multiple reaction monitoring (MRM) transitions and retention indices for 475 endogenous metabolites, enabling rapid and reliable metabolite identification and semi-quantification. Automatic peak identification was carried out with subsequent manual confirmation based on the retention time of the corresponding metabolites present in the database. Any individual metabolite with a coefficient of variation greater than 30% among the QC samples was excluded from further analysis to ensure the reliability of the metabolomic examination.

Data normalization with the peak area of the IS was performed before importing into SimCA 13 (Umetrics AB, Umea, Sweden), where principal component analysis (PCA) was conducted to visualize clustering, trends and outliers on a score plot. Subsequently, the metabolomic data was subjected to partial least square discriminant analysis (PLS-DA) to elucidate the inter-group separation and the metabolites contributing to this differentiation. The PLS-DA model was validated by permutation tests (100 repetitions). Metabolites demonstrating a variable importance in projection (VIP) value > 1 were subjected to a two-tailed unpaired *t*-test with a *p* value of 0.05 to indicate statistical significance. False discovery rates (FDRs) were calculated using the Benjamini–Hochberg procedure to minimize false positive significant results. Metabolites with FDR < 0.05 and VIP > 1 were considered significant and were submitted to MetaboAnalyst (McGill University, Montreal, Quebec, Canada) for pathway analysis.

### 4.7. Statistics

All statistical analyses were performed with GraphPad Prism 8 (GraphPad Software, San Diego, CA, USA). Data are expressed as mean ± standard deviation (SD). The difference between the means of the two groups were analyzed by two-tailed unpaired *t*-test while the difference within the same animal at two different time point was compared by two-tailed paired *t*-test. The difference among more than two groups was analyzed by one-way ANOVA. A *p*-values lesser than 0.05 indicates a statistically significant difference.

## 5. Conclusions

In this study, the health-promoting effects of OXY, ISO and PTS were examined in Sprague Dawley rats, with RES as a comparator. Nontargeted metabolomic analyses were performed using GC-MS/MS. Various in vivo health benefits of these stilbenes were observed, and several key metabolic pathways were found to be modulated by the tested stilbenes. Interestingly, despite their structural similarities, the biological activities of these stilbenes appear to be distinct. In addition to reaffirming the well-known health-promoting effects of RES, this study suggests that future nutraceutical research on ISO, OXY and PTS should focus on Alzheimer’s disease, cardiovascular disease, metabolic syndrome and/or inflammation. Clearly, ISO, OXY and PTS have emerged as promising candidates for further nutraceutical development.

## Data Availability

The data presented in this study are available upon request from the corresponding authors.

## Supplementary Materials

The following supporting information can be downloaded at: https://www.mdpi.com/article/10.3390/ijms252011027/s1.

## Abbreviations

CMC	carboxymethyl cellulose
FBG	fasting blood glucose
FDRs	false discovery rates
GC-MS/MS	gas chromatography–tandem mass spectrometry
IS	internal standard
ISO	isorhapontigenin
KEGG	Kyoto Encyclopedia of Genes and Genomes
MRM	multiple reaction monitoring
Oxy	oxyresveratrol
PCA	principal component analysis
PLS-DA	partial least square discriminant analysis
PPP	pentose phosphate pathway
PTS	pterostilbene
QC	quality control
RES	resveratrol
ROS	reactive oxygen species
TCA	tricarboxylic acid
VIP	variable importance in projection

## References

[1] Singh A.P., Singh R., Verma S.S., Rai V., Kaschula C.H., Maiti P., Gupta S.C. (2019). Health benefits of resveratrol: Evidence from clinical studies. Med. Res. Rev..

[2] Zhang L.X., Li C.X., Kakar M.U., Khan M.S., Wu P.F., Amir R.M., Dai D.F., Naveed M., Li Q.Y., Saeed M. (2021). Resveratrol (RV): A pharmacological review and call for further research. Biomed. Pharmacother..

[3] Dai Y., Tan A.L.C., Chen H., Ong P.S., Xiang X., Wu J., Lin H.S. (2018). Quantification of desoxyrhapontigenin (4-methoxyresveratrol) in rat plasma by LC-MS/MS: Application to pre-clinical pharmacokinetic study. J. Pharm. Biomed. Anal..

[4] Yeo S.C.M., Fenwick P.S., Barnes P.J., Lin H.S., Donnelly L.E. (2017). Isorhapontigenin, a bioavailable dietary polyphenol, suppresses airway epithelial cell inflammation through a corticosteroid-independent mechanism. Br. J. Pharmacol..

[5] Chen W., Yeo S.C.M., Elhennawy M., Lin H.S. (2016). Oxyresveratrol: A bioavailable dietary polyphenol. J. Funct. Food..

[6] Choo Q.Y., Yeo S.C.M., Ho P.C., Tanaka Y., Lin H.S. (2014). Pterostilbene surpassed resveratrol for anti-inflammatory application: Potency consideration and pharmacokinetics perspective. J. Funct. Food..

[7] Pecyna P., Wargula J., Murias M., Kucinska M. (2020). More Than Resveratrol: New Insights into Stilbene-Based Compounds. Biomolecules.

[8] Dai Y., Lim J.X., Yeo S.C.M., Xiang X., Tan K.S., Fu J.H., Huang L., Lin H.S. (2020). Biotransformation of Piceatannol, a Dietary Resveratrol Derivative: Promises to Human Health. Mol. Nutr. Food Res..

[9] Lange K.W., Li S. (2018). Resveratrol, pterostilbene, and dementia. Biofactors.

[10] Yeo S.C., Ho P.C., Lin H.S. (2013). Pharmacokinetics of pterostilbene in Sprague-Dawley rats: The impacts of aqueous solubility, fasting, dose escalation, and dosing route on bioavailability. Mol. Nutr. Food Res..

[11] Dai Y., Yeo S.C.M., Barnes P.J., Donnelly L.E., Loo L.C., Lin H.S. (2018). Pre-clinical Pharmacokinetic and Metabolomic Analyses of Isorhapontigenin, a Dietary Resveratrol Derivative. Front. Pharmacol..

[12] Wishart D.S. (2016). Emerging applications of metabolomics in drug discovery and precision medicine. Nat. Rev. Drug Discov..

[13] Patti G.J., Yanes O., Siuzdak G. (2012). Innovation: Metabolomics: The apogee of the omics trilogy. Nat. Rev. Mol. Cell Biol..

[14] Korsholm A.S., Kjaer T.N., Ornstrup M.J., Pedersen S.B. (2017). Comprehensive Metabolomic Analysis in Blood, Urine, Fat, and Muscle in Men with Metabolic Syndrome: A Randomized, Placebo-Controlled Clinical Trial on the Effects of Resveratrol after Four Months’ Treatment. Int. J. Mol. Sci..

[15] Brennan L. (2019). Metabolomics: A Powerful Tool to Enrich our Understanding of the Impact of Food on Health. Mol. Nutr. Food Res..

[16] Brennan L., Hu F.B. (2019). Metabolomics-Based Dietary Biomarkers in Nutritional Epidemiology-Current Status and Future Opportunities. Mol. Nutr. Food Res..

[17] Baur J.A., Sinclair D.A. (2006). Therapeutic potential of resveratrol: The in vivo evidence. Nat. Rev. Drug Discov..

[18] Mokdad A.H., Ford E.S., Bowman B.A., Dietz W.H., Vinicor F., Bales V.S., Marks J.S. (2003). Prevalence of obesity, diabetes, and obesity-related health risk factors, 2001. JAMA.

[19] Torres Santiago G., Serrano Contreras J.I., Melendez Camargo M.E., Zepeda Vallejo L.G. (2019). NMR-based metabonomic approach reveals changes in the urinary and fecal metabolome caused by resveratrol. J. Pharm. Biomed. Anal..

[20] Wang Y.R., Tsai Y.F., Lau Y.T., Yu H.P. (2015). Plasma metabolite profiles following trauma-hemorrhage: Effect of posttreatment with resveratrol. Shock.

[21] Etxeberria U., Arias N., Boque N., Romo-Hualde A., Macarulla M.T., Portillo M.P., Milagro F.I., Martinez J.A. (2015). Metabolic faecal fingerprinting of trans-resveratrol and quercetin following a high-fat sucrose dietary model using liquid chromatography coupled to high-resolution mass spectrometry. Food Funct..

[22] Phua L.C., Wilder-Smith C.H., Tan Y.M., Gopalakrishnan T., Wong R.K., Li X., Kan M.E., Lu J., Keshavarzian A., Chan E.C. (2015). Gastrointestinal Symptoms and Altered Intestinal Permeability Induced by Combat Training Are Associated with Distinct Metabotypic Changes. J. Proteome Res..

[23] Bacik J.P., Klesmith J.R., Whitehead T.A., Jarboe L.R., Unkefer C.J., Mark B.L., Michalczyk R. (2015). Producing glucose 6-phosphate from cellulosic biomass: Structural insights into levoglucosan bioconversion. J. Biol. Chem..

[24] Goodman H.M. (1988). The Role of Growth Hormone in Fat Mobilization.

[25] Saponaro C., Gaggini M., Carli F., Gastaldelli A. (2015). The subtle balance between lipolysis and lipogenesis: A critical point in metabolic homeostasis. Nutrients.

[26] Kruger N.J., Von Schaewen A. (2003). The oxidative pentose phosphate pathway: Structure and organisation. Curr. Opin. Plant Biol..

[27] Balendiran G.K., Dabur R., Fraser D. (2004). The role of glutathione in cancer. Cell Biochem. Funct..

[28] Li S., Tan H.Y., Wang N., Zhang Z.J., Lao L., Wong C.W., Feng Y. (2015). The role of oxidative stress and antioxidants in liver diseases. Int. J. Mol. Sci..

[29] Slyshenkov V.S., Dymkowska D., Wojtczak L. (2004). Pantothenic acid and pantothenol increase biosynthesis of glutathione by boosting cell energetics. FEBS Lett..

[30] Pegg A.E. (1970). Biosynthesis of Putrescine and Polyamines in Mammalian Tissues. Ann. N. Y. Acad. Sci..

[31] Yue F., Li W., Zou J., Jiang X., Xu G., Huang H., Liu L. (2017). Spermidine prolongs lifespan and prevents liver fibrosis and hepatocellular carcinoma by activating MAP1S-mediated autophagy. Cancer Res..

[32] Zhang Z., Jin J., Shi L. (2008). Protective function of cis-mulberroside A and oxyresveratrol from Ramulus mori against ethanol-induced hepatic damage. Environ. Toxicol. Pharmacol..

[33] Degli Esposti D., Hamelin J., Bosselut N., Saffroy R., Sebagh M., Pommier A., Martel C., Lemoine A. (2012). Mitochondrial roles and cytoprotection in chronic liver injury. Biochem. Res. Int..

[34] Chu X.Y., Yang S.Z., Zhu M.Q., Zhang D.Y., Shi X.C., Xia B., Yuan Y., Liu M., Wu J.W. (2020). Isorhapontigenin Improves Diabetes in Mice via Regulating the Activity and Stability of PPARgamma in Adipocytes. J. Agric. Food Chem..

[35] Clark L.T. (1986). Cholesterol and heart disease: Current concepts in pathogenesis and treatment. J. Natl. Med. Assoc..

[36] Doenst T., Nguyen Tien D., Abel E.D. (2013). Cardiac Metabolism in Heart Failure. Circ. Res..

[37] Opie L.H., Knuuti J. (2009). The Adrenergic-Fatty Acid Load in Heart Failure. J. Am. Coll. Cardiol..

[38] Hung L.-M., Chen J.-K., Huang S.-S., Lee R.-S., Su M.-J. (2000). Cardioprotective effect of resveratrol, a natural antioxidant derived from grapes. Cardiovasc. Res..

[39] Tsutsui H., Kinugawa S., Matsushima S. (2011). Oxidative stress and heart failure. Am. J. Physiol. Heart Circ. Physiol..

[40] Kosuru R., Cai Y., Kandula V., Yan D., Wang C., Zheng H., Li Y., Irwin M.G., Singh S., Xia Z. (2018). AMPK Contributes to Cardioprotective Effects of Pterostilbene Against Myocardial Ischemia-Reperfusion Injury in Diabetic Rats by Suppressing Cardiac Oxidative Stress and Apoptosis. Cell. Physiol. Biochem. Int. J. Exp. Cell. Physiol. Biochem. Pharmacol..

[41] Reginaldo C.D., Selhub J., Paul L., Jacques P., Wang T., Gerszten R. (2013). Anthranilic acid and 3-hydroxyanthranilic acid, but not kynurenic acid, are associated with plasma pyridoxal-5 phosphate levels. FASEB J..

[42] Kim J.H., Kim J., Kim H.J., Sathiyanarayanan G., Bhatia S.K., Song H.S., Choi Y.K., Kim Y.G., Park K., Yang Y.H. (2017). Biotransformation of pyridoxal 5′-phosphate from pyridoxal by pyridoxal kinase (pdxY) to support cadaverine production in Escherichia coli. Enzym. Microb. Technol..

[43] Litalien C., Beaulieu P. (2011). Molecular Mechanisms of Drug Actions: From Receptors to Effectors.

[44] Niklasson F., Agren H. (1984). Brain energy metabolism and blood-brain barrier permeability in depressive patients: Analyses of creatine, creatinine, urate, and albumin in CSF and blood. Biol. Psychiatry.

[45] Kamata Y., Shiraga H., Tai A., Kawamoto Y., Gohda E. (2007). Induction of neurite outgrowth in PC12 cells by the medium-chain fatty acid octanoic acid. Neuroscience.

[46] Singh M. (2005). Essential fatty acids, DHA and human brain. Indian. J. Pediatr..

[47] Love S. (1999). Oxidative Stress in Brain Ischemia. Brain Pathol..

[48] Chao J., Yu M.-S., Ho Y.-S., Wang M., Chang R.C.-C. (2008). Dietary oxyresveratrol prevents parkinsonian mimetic 6-hydroxydopamine neurotoxicity. Free Radic. Biol. Med..

[49] National Institutes of Health Higher Brain Glucose Levels May Mean More Severe Alzheimer’s. https://www.nih.gov/news-events/news-releases/higher-brain-glucose-levels-may-mean-more-severe-alzheimers.

[50] Jeon S.Y., Kwon S.H., Seong Y.H., Bae K., Hur J.M., Lee Y.Y., Suh D.Y., Song K.S. (2007). β-secretase (BACE1)-inhibiting stilbenoids from Smilax Rhizoma. Phytomedicine.

[51] Ryou M.-G., Liu R., Ren M., Sun J., Mallet Robert T., Yang S.-H. (2012). Pyruvate Protects the Brain against Ischemia–Reperfusion Injury by Activating the Erythropoietin Signaling Pathway. Stroke.

[52] Hong C.T., Chau K.-Y., Schapira A.H.V. (2016). Meclizine-induced enhanced glycolysis is neuroprotective in Parkinson disease cell models. Sci. Rep..

[53] Chaturvedi R.K., Beal M.F. (2008). Mitochondrial Approaches for Neuroprotection. Ann. N. Y. Acad. Sci..

[54] Oja S.S., Saransaari P. (1996). Taurine as osmoregulator and neuromodulator in the brain. Metab. Brain Dis..

[55] Mokni M., Elkahoui S., Limam F., Amri M., Aouani E. (2007). Effect of resveratrol on antioxidant enzyme activities in the brain of healthy rat. Neurochem. Res..

[56] Ates O., Cayli S., Altinoz E., Gurses I., Yucel N., Sener M., Kocak A., Yologlu S. (2007). Neuroprotection by resveratrol against traumatic brain injury in rats. Mol. Cell. Biochem..

[57] Chang J., Rimando A., Pallas M., Camins A., Porquet D., Reeves J., Shukitt-Hale B., Smith M.A., Joseph J.A., Casadesus G. (2012). Low-dose pterostilbene, but not resveratrol, is a potent neuromodulator in aging and Alzheimer’s disease. Neurobiol. Aging.

[58] Movahed A., Movahed A., Nabipour I., Louis X.L., Thandapilly S.J., Yu L., Kalantarhormozi M., Rekabpour S.J., Netticadan T. (2017). Antihyperglycemic Effects of Short Term Resveratrol Supplementation in Type 2 Diabetic Patients Antihyperglycemic Effects of Short Term Resveratrol Supplementation in Type 2 Diabetic Patients. Hindawi.

[59] Moghei M., Tavajohi-Fini P., Beatty B., Adegoke O.A.J. (2016). Ketoisocaproic acid, a metabolite of leucine, suppresses insulin-stimulated glucose transport in skeletal muscle cells in a BCAT2-dependent manner. Am. J. Physiol. Cell Physiol..

[60] Alayev A., Berger S.M., Holz M.K. (2015). Resveratrol as a novel treatment for diseases with mTOR pathway hyperactivation. Ann. N. Y. Acad. Sci..

[61] Park D., Noh J., Ryu S.H., Park H., Yang Y.R., Suh P.-G., Kwon O., Jeong H., Koh A., Lee M.N. (2016). Resveratrol induces autophagy by directly inhibiting mTOR through ATP competition. Sci. Rep..

[62] Arner P., Rydén M. (2015). Fatty acids, obesity and insulin resistance. Obes. Facts.

[63] Madeo F., Eisenberg T., Pietrocola F., Kroemer G. (2018). Spermidine in health and disease. Science.

[64] Moreth K., Gailus-Durner V., Trausinger G., Sedej S., Pieber T., Stekovic S., Moustafa T., Meinitzer A., Dammbrueck C., Zimmermann A. (2016). Cardioprotection and lifespan extension by the natural polyamine spermidine. Nat. Med..

[65] Zhuang P., Shou Q., Lu Y., Wang G., Qiu J., Wang J., He L., Chen J., Jiao J., Zhang Y. (2017). Arachidonic acid sex-dependently affects obesity through linking gut microbiota-driven inflammation to hypothalamus-adipose-liver axis. Biochim. Et. Biophys. Acta—Mol. Basis Dis..

[66] Zhao X., Han Q., Liu Y., Sun C., Gang X., Wang G. (2016). The Relationship between Branched-Chain Amino Acid Related Metabolomic Signature and Insulin Resistance: A Systematic Review. J. Diabetes Res..

[67] McCormack S.E., Shaham O., McCarthy M.A., Deik A.A., Wang T.J., Gerszten R.E., Clish C.B., Mootha V.K., Grinspoon S.K., Fleischman A. (2013). Circulating Branched-chain Amino Acid Concentrations Are Associated with Obesity and Future Insulin Resistance in Children and Adolescents. Pediatr. Obes..

[68] Lunt S.Y., Vander Heiden M.G. (2011). Aerobic Glycolysis: Meeting the Metabolic Requirements of Cell Proliferation. Annu. Rev. Cell Dev. Biol..

[69] Berg J.M., Tymoczko J.L., Stryer L. (2002). Chapter 16 Glycolysis and Gluconeogenesis, Biochemistry.

[70] Dahlqvist A., Thomson D.L. (1963). The digestion and absorption of sucrose by the intact rat. Acta Physiol..

[71] Kilkenny C., Browne W.J., Cuthill I.C., Emerson M., Altman D.G. (2010). Improving bioscience research reporting: The ARRIVE guidelines for reporting animal research. PLoS Biol..

[72] Li X., Yang J., Jin S., Dai Y., Fan Y., Fan X., Li Z., Yang J., Yau W.P., Lin H. (2020). Mechanistic examination of methimazole-induced hepatotoxicity in patients with Grave’s disease: A metabolomic approach. Arch. Toxicol..

[73] Kong S.T., Lin H.S., Ching J., Xie H., Ho P.C. (2022). Dried Blood Spots as Matrix for Evaluation of Valproate Levels and the Immediate and Delayed Metabolomic Changes Induced by Single Valproate Dose Treatment. Int. J. Mol. Sci..

