# Supplementary material for: Exploration of Nutraceutical Potentials of Isorhapontigenin, Oxyresveratrol and Pterostilbene: A Metabolomic Approach
Source: Int J Mol Sci. 2024 Oct 14;25(20):11027. doi: 10.3390/ijms252011027 (PMC11507072; doi:10.3390/ijms252011027)

Supplementary Table S1. Fold change of statistically significant hepatic metabolites after 2-week RES, PTS, OXY and ISO intervention.

Resveratrol				
**Metabolites**	***p*-value**	**FDR**	**VIP**	**Fold change**
Glyceric acid	0.002	0.044	1.742	1.673
2-Ketoisocaproic acid	0.003	0.044	1.901	1.712
Nicotinic acid	0.003	0.044	1.668	0.666
1,6-Anhydroglucose	0.003	0.044	1.537	0.688
3-Phosphoglyceric acid	0.005	0.047	2.488	2.293
Glucose 6-phosphate	0.006	0.047	1.493	1.32
**Pterostilbene**				
**Metabolites**	***p*-value**	**FDR**	**VIP**	**Fold change**
Glyceric acid	<0.001	<0.001	2.169	1.915
Palmitic acid	<0.001	0.008	1.184	1.257
Fructose 1-phosphate	<0.001	0.011	2.688	0.374
Spermidine	0.001	0.019	1.942	0.529
Arachidonic acid	0.002	0.024	1.231	1.229
Linoleic acid	0.003	0.024	1.302	1.374
Fructose 6-phosphate	0.003	0.024	2.005	0.547
Myristic acid	0.003	0.024	1.595	1.597
3-Aminoisobutyric acid	0.004	0.03	2.062	0.454
Niacinamide	0.007	0.04	1.571	0.613
5-Methoxytryptamine	0.007	0.04	1.526	0.657
Elaidic acid	0.008	0.04	1.032	1.256
Oleic acid	0.009	0.042	1.114	1.309
Epinephrine	0.009	0.042	1.642	0.566
**Oxyresveratrol**				
**Metabolites**	***p*-value**	**FDR**	**VIP**	**Fold change**
2-Aminoisobutyric acid	<0.001	<0.001	1.458	1.419
Glucaric acid	<0.001	0.004	1.989	0.519
Xylitol	<0.001	0.004	1.463	0.676
Epinephrine	<0.001	0.004	1.757	1.778
Arabitol	<0.001	0.004	1.461	0.676
Ribonolactone	<0.001	0.006	1.375	0.705
Ornithine	<0.001	0.009	1.718	1.901
Margaric acid	0.001	0.009	1.186	1.336
Octadecanol	0.001	0.009	1.156	1.318
Threo-b-hydroxyaspartic acid	0.002	0.01	1.433	1.486
Pantothenic acid	0.002	0.015	1.009	1.268
Lyxose	0.004	0.02	1.258	0.687
3-Hydroxyisovaleric acid	0.005	0.025	1.013	1.258
Glycyl-Glycine	0.006	0.025	1.133	1.345

1,6-Anhydroglucose	0.006	0.025	1.216	0.715
Putrescine	0.008	0.035	1.07	1.323
Ribulose 5-phosphate	0.012	0.043	1.925	2.078
Glucuronic acid	0.012	0.043	1.175	0.701
Galacturonic acid	0.013	0.043	1.164	0.701
2-Deoxy-glucose	0.013	0.043	1.17	0.713
Ribose	0.014	0.043	1.254	1.365
2-Ketoisocaproic acid	0.015	0.043	1.454	1.503
Myristic acid	0.015	0.043	1.14	1.406
Ribose 5-phosphate	0.016	0.044	1.387	1.607
**Isorhapontigenin**				
**Metabolites**	***p*-value**	**FDR**	**VIP**	**Fold change**
2-Phosphoglyceric acid	<0.001	<0.001	2.353	2.742
3-Phosphoglyceric acid	<0.001	<0.001	2.511	3.241
Spermidine	<0.001	<0.001	1.697	1.805
Glycyl-Glycine	<0.001	<0.001	1.402	1.497
2-Ketoisocaproic acid	<0.001	0.001	1.804	1.975
Cadaverine	<0.001	0.003	1.337	1.476
Linoleic acid	<0.001	0.003	1.162	1.31
Glucose 6-phosphate	<0.001	0.003	1.441	1.635
Dopamine	<0.001	0.005	1.229	1.422
Dihydrouracil	<0.001	0.005	1.141	1.362
3-Hydroxyisovaleric acid	0.001	0.007	1.271	1.377
Glyceric acid	0.002	0.008	1.424	1.639
Octopamine	0.002	0.008	1.467	1.723
2-Aminoisobutyric acid	0.002	0.009	1.225	1.401
Tyramine	0.003	0.009	1.336	1.598
Fructose	0.003	0.011	1.288	1.515
Citric acid	0.004	0.012	1.043	1.332
Epinephrine	0.004	0.012	1.339	1.598
Ribonic acid	0.005	0.015	1.276	1.497
Putrescine	0.005	0.015	1.148	1.32
Spermine	0.006	0.016	1.921	2.239
Ribulose	0.007	0.017	1.069	1.377
Fructose 1-phosphate	0.008	0.021	1.214	1.47
Succinic acid	0.009	0.021	1.649	2.107
Xylulose	0.009	0.021	1.05	1.362
Glucaric acid	0.01	0.022	1.013	0.765
2-Hydroxyglutaric acid	0.01	0.022	1.25	1.597
Ribose	0.014	0.028	1.129	1.444
Threo-b-hydroxyaspartic acid	0.019	0.036	1.094	1.462
Fructose 6-phosphate	0.019	0.036	1.082	1.401
Ribulose 5-phosphate	0.023	0.041	1.52	2.038

2-Ketoglutaric acid	0.025	0.044	1.492	2.027
Adipic acid	0.026	0.044	1.032	1.325
Uridine	0.028	0.047	1.753	2.340

Supplementary Table S2. Fold change of statistically significant cardiac metabolites after 2-week RES, PTS, OXY and ISO intervention.

**Resveratrol**				
**Metabolites**	***p*-value**	**FDR**	**VIP**	**Fold change**
Octanoic acid	<0.001	0.026	2.296	0.446
1,6-Anhydroglucose	0.002	0.033	1.607	0.643
Glucosamine	0.002	0.033	1.524	0.670
Caproic acid	0.002	0.033	2.139	0.480
Succinic acid	0.005	0.046	1.230	0.752
2-Hydroxyglutaric acid	0.005	0.046	1.957	0.524
Galacturonic acid	0.005	0.046	2.196	0.540
Biotin	0.006	0.046	1.550	0.637
Glucuronic acid	0.006	0.046	2.297	0.492
Sedoheptulose 7-phosphate	0.007	0.047	1.678	0.592
2-Aminoisobutyric acid	0.008	0.048	1.143	0.796
**Pterostilbene**				
**Metabolites**	***p*-value**	**FDR**	**VIP**	**Fold change**
Galacturonic acid	<0.001	0.003	2.091	0.442
2-Aminoisobutyric acid	0.001	0.009	1.330	0.704
2-Hydroxyglutaric acid	0.001	0.009	2.166	0.395
Octanoic acid	0.001	0.009	1.937	0.477
3-Sulfinoalanine	0.001	0.009	2.752	0.261
Glucuronic acid	0.001	0.009	2.226	0.387
Kynurenine	0.001	0.009	1.461	0.640
3-Aminopropanoic acid	0.001	0.010	1.648	0.579
Glucosamine	0.001	0.011	1.402	0.654
Acetoacetic acid	0.002	0.012	1.612	0.585
Lyxose	0.003	0.018	1.355	0.666
Ribulose	0.004	0.019	1.121	1.335
Galactose	0.004	0.019	1.208	0.713
Mannose	0.005	0.019	1.208	0.715
Glucose	0.005	0.019	1.195	0.719
Margaric acid	0.005	0.019	1.218	0.689
4-Aminobutyric acid	0.005	0.019	1.869	0.504
Psicose	0.005	0.019	1.223	0.707
Ureidosuccinic acid	0.006	0.019	1.962	0.416
Xylulose	0.006	0.019	1.108	1.332

3-Hydroxypropionic acid	0.007	0.023	1.168	0.721
Ribose	0.007	0.023	1.101	1.345
Maleic acid	0.010	0.029	1.337	0.632
Caproic acid	0.011	0.031	1.486	0.601
Niacinamide	0.012	0.034	1.037	0.745
Glycyl-Glycine	0.013	0.034	1.336	0.620
2-Ketoglutaric acid	0.013	0.034	1.266	0.630
Anthranilic acid	0.014	0.036	1.234	1.319
Pyridoxal	0.015	0.037	1.141	0.752
Biotin	0.016	0.037	1.127	0.698
**Oxyresveratrol**				
**Metabolites**	***p*-value**	**FDR**	**VIP**	**Fold change**
Galacturonic acid	<0.001	0.010	2.505	0.401
Octanoic acid	0.001	0.017	2.091	0.486
Caproic acid	0.001	0.017	2.404	0.409
Glucosamine	0.001	0.017	1.734	0.622
Pyridoxal	0.002	0.020	1.498	0.704
1,6-Anhydroglucose	0.002	0.024	1.540	0.661
Psicose	0.003	0.024	1.578	0.652
Glyceraldehyde	0.004	0.033	2.115	0.428
Lyxose	0.007	0.047	1.576	0.662
Cholesterol	0.010	0.047	1.265	0.724
Mannose	0.011	0.047	1.242	0.740
Glucose	0.011	0.047	1.226	0.745
Ascorbic acid	0.012	0.047	1.278	0.726
Galactose	0.012	0.047	1.211	0.747
Kynurenine	0.012	0.047	1.615	0.647
Sedoheptulose 7-phosphate	0.013	0.047	1.444	0.639
Glucuronic acid	0.013	0.047	2.370	0.430
Margaric acid	0.014	0.047	1.137	0.747
**Isorhapontigenin**				
**Metabolites**	***p*-value**	**FDR**	**VIP**	**Fold change**
3-Hydroxyisovaleric acid	<0.001	<0.001	1.567	0.637
Galacturonic acid	<0.001	0.001	2.324	0.351
Glucuronic acid	<0.001	0.004	2.431	0.282
2-Aminoisobutyric acid	<0.001	0.004	1.293	0.687
Octanoic acid	<0.001	0.004	2.033	0.419
Galactitol	<0.001	0.004	1.130	0.728
2-Hydroxyglutaric acid	0.001	0.006	2.205	0.381
Ribose	0.001	0.009	1.130	1.329
Caproic acid	0.001	0.009	2.015	0.412
Acetoacetic acid	0.001	0.009	1.521	0.587

3-Sulfinoalanine	0.002	0.012	2.151	0.350
Xylulose	0.002	0.012	1.076	1.326
Ribulose	0.002	0.012	1.075	1.326
2-Ketoisocaproic acid	0.003	0.014	1.672	0.495
Threitol	0.003	0.014	1.152	0.702
Kynurenine	0.004	0.017	1.275	0.668
Lyxose	0.004	0.017	1.248	0.678
Dopamine	0.005	0.018	1.993	0.377
Biotin	0.005	0.018	1.385	0.605
Ureidosuccinic acid	0.007	0.021	1.817	0.430
Sedoheptulose 7-phosphate	0.007	0.021	1.420	0.579
Pyridoxal	0.008	0.021	1.132	0.724
Octopamine	0.008	0.021	1.232	0.640
3-Hydroxypropionic acid	0.008	0.021	1.094	0.725
Margaric acid	0.008	0.022	1.080	0.706
2-Ketoglutaric acid	0.012	0.031	1.229	0.624
Erythritol	0.012	0.031	1.293	0.636
Spermidine	0.015	0.035	1.355	0.577
Niacinamide	0.017	0.039	1.029	0.727
Norepinephrine	0.017	0.039	1.174	0.695
Anthranilic acid	0.018	0.040	1.010	1.297
Tryptamine	0.022	0.045	1.239	0.614
Ribitol	0.022	0.045	1.102	0.692
Norvaline	0.025	0.049	1.085	0.692
Maleic acid	0.025	0.049	1.154	0.677

Supplementary Table S3. Fold change of statistically significant brain metabolites after 2-week RES, PTS, OXY and ISO intervention.

**Resveratrol**				
**Metabolites**	***p*-value**	**FDR**	**VIP**	**Fold change**
Arachidonic acid	<0.001	0.002	1.014	0.832
Eicosapentaenoic acid	<0.001	0.002	1.024	0.829
Glyceraldehyde 3-phosphate	<0.001	0.005	2.094	0.453
5-Aminovaleric acid	<0.001	0.005	2.198	0.421
2-Ketoisocaproic acid	<0.001	0.005	1.895	1.872
Erythrose 4-phosphate	<0.001	0.005	1.896	0.520
Erythrulose	0.001	0.005	2.461	2.660
Dihydrouracil	0.001	0.011	2.213	0.520
N-Acetylneuraminic acid	0.002	0.011	1.706	0.561
Linoleic acid	0.002	0.011	1.382	1.346
Glycerol 2-phosphate	0.002	0.014	1.436	0.676
Niacinamide	0.003	0.018	1.202	0.794
Oleic acid	0.004	0.019	1.035	0.781

Creatinine	0.004	0.020	1.946	0.542
2-Aminoisobutyric acid	0.005	0.020	1.253	1.246
Caproic acid	0.005	0.020	1.215	1.408
Ribose	0.007	0.027	1.694	1.694
Glutamine	0.009	0.032	1.395	0.663
Ornithine	0.009	0.032	1.624	0.577
Octopamine	0.010	0.035	2.043	0.370
Serine	0.011	0.035	1.128	0.738
**Pterostilbene**				
**Metabolites**	***p*-value**	**FDR**	**VIP**	**Fold change**
Glutamic acid	0.001	0.036	1.476	1.551
Erythrulose	0.001	0.036	1.901	1.822
Pyruvic acid	0.002	0.037	1.548	1.465
Caproic acid	0.003	0.037	1.566	1.456
Ribose	0.003	0.037	1.273	1.383
Threitol	0.004	0.037	1.408	1.344
Adipic acid	0.005	0.037	1.092	0.705
2-Ketoadipic acid	0.006	0.037	1.666	1.573
Sorbose	0.007	0.037	1.315	0.718
Tagatose	0.007	0.037	1.315	0.716
Psicose	0.008	0.037	1.291	0.729
Allose	0.008	0.037	1.755	0.601
Galactose	0.009	0.037	1.722	0.599
Mannose	0.009	0.037	1.768	0.583
Citramalic acid	0.010	0.039	1.157	1.249
Norvaline	0.012	0.043	1.607	1.673
Fructose	0.013	0.044	1.279	0.715
Glutamine	0.015	0.044	1.063	1.305
N-Acetylaspartic acid	0.015	0.044	1.883	1.552
Xylose	0.016	0.044	1.294	1.248
Ornithine	0.016	0.044	1.404	0.722
3-Phosphoglyceric acid	0.018	0.044	2.664	2.939
Lyxose	0.019	0.044	1.268	1.238
Glyceraldehyde 3-phosphate	0.020	0.044	1.482	0.685
Ribose 5-phosphate	0.021	0.044	1.155	1.308
O-Phosphoethanolamine	0.022	0.044	1.428	1.378
3-Hydroxypyruvic acid	0.022	0.044	1.360	1.566
Glyceric acid	0.023	0.044	1.637	1.630
Arabinose	0.023	0.044	1.243	1.238
Tyramine	0.023	0.044	1.297	1.415
Succinic acid	0.025	0.045	1.027	1.213
Glucose	0.025	0.045	1.616	0.616
2-Phosphoglyceric acid	0.026	0.045	2.647	2.944

Threonic acid	0.028	0.048	1.238	1.310

Oxyresveratrol

Metabolites	*p*-value	FDR	VIP	Fold change
Glutamine	<0.001	0.011	1.541	0.690
Ribose	0.001	0.011	1.898	1.723
Sorbose	0.001	0.011	1.665	0.573
Caproic acid	0.001	0.011	1.783	1.744
Psicose	0.001	0.011	1.553	0.618
Tagatose	0.002	0.011	1.603	0.605
Fructose	0.002	0.011	1.681	0.570
Ornithine	0.002	0.011	1.560	0.555
Glyceraldehyde 3-phosphate	0.003	0.014	1.599	0.572
Mannose	0.003	0.015	1.811	0.499
Glucose	0.004	0.015	1.750	0.532
Allose	0.004	0.015	1.845	0.504
Galactose	0.004	0.015	1.800	0.515
Fructose 6-phosphate	0.004	0.015	1.849	0.530
3-Phosphoglyceric acid	0.004	0.015	2.343	2.742
Mannose	0.005	0.015	1.815	0.490
2-Phosphoglyceric acid	0.005	0.015	2.286	2.564
Mannose 6-phosphate	0.005	0.015	1.784	0.545
Glucose 6-phosphate	0.008	0.020	1.718	0.564
Oleic acid	0.008	0.020	1.045	0.796
Pantothenic acid	0.008	0.020	1.203	1.406
Tryptamine	0.009	0.022	1.219	1.429
Fructose 1-phosphate	0.010	0.022	1.730	0.568
Erythrulose	0.011	0.022	2.028	2.086
Creatinine	0.012	0.024	1.435	0.544
Glycerol 2-phosphate	0.013	0.025	1.410	0.744
Pyruvic acid	0.013	0.025	1.138	1.300
Glyceric acid	0.014	0.025	1.299	1.493
Ribitol	0.018	0.033	1.473	1.596
Phenylalanine	0.019	0.034	1.827	0.369
Sedoheptulose 7-phosphate	0.020	0.034	1.656	0.601
Maltose	0.022	0.036	1.107	0.771
Methionine	0.024	0.039	1.728	0.396
Arabitol	0.030	0.045	1.004	1.461
3-Aminoglutaric acid	0.031	0.045	1.619	0.508

Isorhapontigenin

Metabolites	*p*-value	FDR	VIP	Fold change
Dihydrouracil	<0.001	0.002	2.030	0.410
3-Aminoglutaric acid	<0.001	0.005	2.421	0.289

Creatinine	<0.001	0.004	2.073	0.371
Glutamine	<0.001	0.003	1.740	0.504
Ornithine	<0.001	0.004	1.808	0.464
Aspartic acid	<0.001	0.004	2.367	0.301
Oleic acid	0.001	0.012	1.372	0.629
Glycerol 2-phosphate	0.002	0.017	1.212	0.687
Glutaric acid	0.003	0.018	1.403	1.690
2-Deoxy-glucose	0.004	0.021	1.606	0.485
Niacinamide	0.005	0.026	1.704	0.518
Phenylalanine	0.005	0.025	2.593	0.212
Linoleic acid	0.005	0.024	1.949	2.363
Methionine	0.006	0.024	2.669	0.231
Lauric acid	0.007	0.026	1.042	0.734
Cadaverine	0.008	0.028	1.861	0.378
Batyl alcohol	0.008	0.029	1.319	0.634
Fructose 6-phosphate	0.011	0.035	1.415	0.564
Glutamic acid	0.011	0.035	1.780	0.511
Taurine	0.012	0.035	2.153	0.286
Elaidic acid	0.016	0.045	1.045	0.741
Tryptamine	0.018	0.047	1.089	1.412
Glycyl-Glycine	0.018	0.045	1.449	0.503
Serine	0.019	0.047	1.750	0.485
Fructose	0.020	0.046	1.028	0.710
Allose	0.020	0.046	1.149	0.652

Supplementary Table S4. Fold change of statistically significant plasma metabolites after 2-week RES, PTS, OXY and ISO intervention.

Resveratrol				
**Metabolites**	***p*-value**	**FDR**	**VIP**	**Fold change**
Spermidine	<0.001	0.006	2.092	2.707
Glutamic acid	<0.001	0.013	2.689	3.43
Ribulose	0.001	0.013	1.643	2.308
Glycolic acid	0.001	0.013	1.292	0.683
Xylulose	0.001	0.013	1.661	2.394
Tyrosine	0.002	0.013	1.801	2.237
4-Aminobutyric acid	0.002	0.013	1.76	2.236
Ribose	0.003	0.016	2.094	3.484
Methionine	0.003	0.016	2.363	3.754
Dihydrouracil	0.004	0.016	1.253	1.597
Glycerol	0.004	0.017	1.348	0.603
Tyramine	0.005	0.018	1.738	2.04
2-Ketoisocaproic acid	0.005	0.019	1.389	0.588

3-Methyl-2-oxovaleric acid	0.006	0.019	1.162	0.657
Anthranilic acid	0.007	0.021	1.423	1.936
Phenylalanine	0.009	0.025	1.944	2.231
N-Acetylmannosamine	0.01	0.026	1.188	0.618
4-Hydroxyproline	0.01	0.026	1.867	2.515
Glycerol 2-phosphate	0.012	0.029	1.14	1.619
Glucono-1,4-lactone	0.019	0.041	1.414	0.593
Trehalose	0.022	0.041	2.762	7.585
Maltose	0.022	0.041	2.769	7.767
Cytidine	0.023	0.041	2.695	7.724
Lactitol	0.023	0.041	2.253	5.502
Rhamnose	0.024	0.041	1.118	1.589
Ascorbic acid	0.024	0.041	1.704	1.989
Batyl alcohol	0.024	0.041	2.58	6.376
3-Hydroxy-3-methylglutaric acid	0.025	0.041	1.057	1.542
Malic acid	0.026	0.041	1.427	1.689
N-Acetylserine	0.029	0.044	1.161	1.546
Pyridoxal	0.032	0.047	1.566	0.498
Glucaric acid	0.035	0.05	1.412	0.59
**Pterostilbene**				
**Metabolites**	***p*-value**	**FDR**	**VIP**	**Fold change**
Monostearin	<0.001	<0.001	2.979	8.713
2-Aminoisobutyric acid	<0.001	<0.001	2.303	0.254
3-Hydroxypropionic acid	<0.001	0.001	1.01	0.742
Dihydrouracil	<0.001	0.001	1.372	1.683
Glycerol	<0.001	0.003	1.351	0.585
Isoleucine	<0.001	0.003	1.338	0.576
3-Methyl-2-oxovaleric acid	<0.001	0.003	1.374	0.561
Methylsuccinic acid	<0.001	0.003	1.619	0.486
Phosphoric acid	<0.001	0.004	1.038	0.728
Glutamic acid	<0.001	0.004	2.54	3.213
N-Acetylmannosamine	<0.001	0.004	1.807	0.393
Ornithine	0.001	0.005	1.235	0.611
Spermidine	0.002	0.007	1.795	2.52
Fumaric acid	0.002	0.009	1.464	0.491
Sorbose	0.003	0.01	1.111	1.549
Urea	0.003	0.011	1.109	1.514
Glucaric acid	0.004	0.012	1.751	0.426
Methionine	0.007	0.019	2.079	3.204
Glycerol 2-phosphate	0.009	0.024	1.112	1.568
Succinic acid	0.011	0.025	1.694	0.374
Cytidine	0.011	0.025	2.47	6.95
Trehalose	0.011	0.025	2.582	7.453

Maltose	0.011	0.025	2.57	7.528
Lactitol	0.011	0.025	1.993	5.396
Ribonolactone	0.014	0.027	1.091	0.622
Batyl alcohol	0.014	0.027	2.346	5.99
N-Acetylserine	0.014	0.027	1.165	1.638
Glucono-1,4-lactone	0.014	0.027	1.429	0.539
2-Ketoisocaproic acid	0.015	0.027	1.086	0.639
Nicotinic acid	0.017	0.031	1.093	0.671
Phenylacetic acid	0.019	0.033	1.263	0.536
Lactic acid	0.02	0.033	1.195	0.59
Norepinephrine	0.023	0.037	1.068	0.603
Acetoacetic acid	0.023	0.037	1.085	1.575
Anthranilic acid	0.031	0.044	1.095	1.559
Eicosapentaenoic acid	0.032	0.045	1.056	0.699
Glutaric acid	0.035	0.048	1.112	0.556
Arachidonic acid	0.036	0.048	1.045	0.704
Isocitric acid	0.038	0.05	1.228	0.504
**Isorhapontigenin**				
**Metabolites**	***p*-value**	**FDR**	**VIP**	**Fold change**
Spermidine	0.001	0.019	2.383	3.208
Xylitol	0.001	0.019	2.063	0.395
Ribonolactone	0.001	0.019	1.925	0.444
Glucaric acid	0.005	0.041	1.886	0.446
Methylsuccinic acid	0.006	0.041	1.343	0.653
Glycolic acid	0.007	0.041	1.856	2.456
Glucono-1,4-lactone	0.008	0.041	1.581	0.531
4-Hydroxyproline	0.01	0.041	1.98	2.464
Methionine	0.01	0.041	2.149	2.596
Glycerol 2-phosphate	0.01	0.041	1.097	1.348
Isoleucine	0.016	0.041	1.143	0.698
Cytidine	0.016	0.041	3.176	8.309
Urea	0.016	0.041	1.017	1.332
Batyl alcohol	0.017	0.041	3.045	6.851
Lactitol	0.018	0.041	2.652	5.382
Maltose	0.019	0.041	3.185	8.033
Gluconic acid	0.019	0.041	1.221	0.657
Pyruvic acid	0.019	0.041	2.281	0.275
Trehalose	0.02	0.041	3.168	7.789
Elaidic acid	0.02	0.041	1.026	1.403
Lactic acid	0.022	0.043	1.313	0.6
Succinic acid	0.024	0.044	1.552	0.477

Supplementary Figure S1. PLS-DA scores plot comparing (A) RES, (C) PTS, (E) OXY and (G) ISO with vehicle in heart. Permutation graphs for PLS-DA model in cardiac samples: (B) RES, (D) PTS, (F) OXY and (H) ISO.

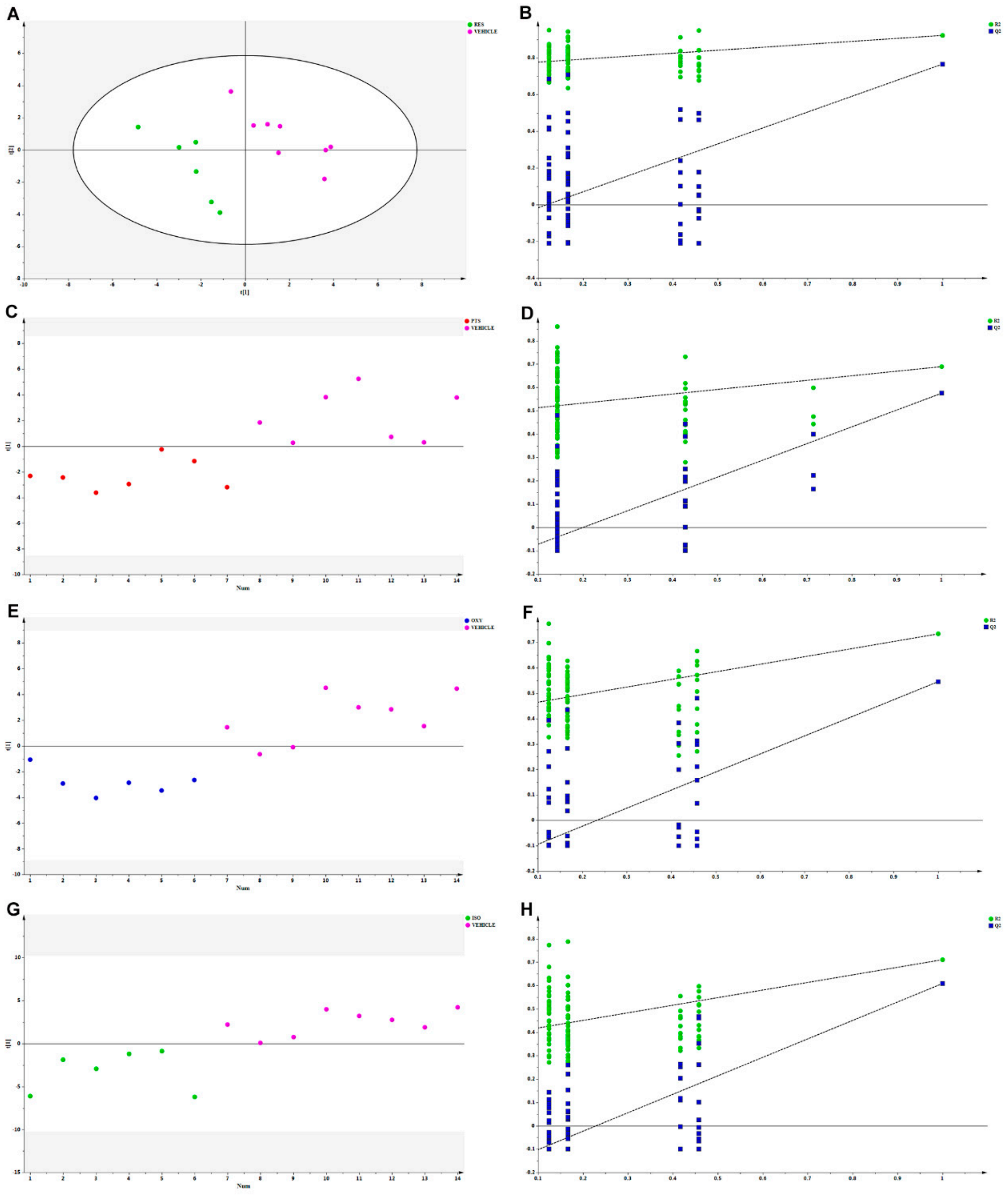

Supplementary Figure S2. PLS-DA scores plot comparing (A) RES, (C) PTS, (E) OXY and (G) ISO with vehicle in brain. Permutation graphs for PLS-DA model in brain samples: (B) RES, (D) PTS, (F) OXY and (H) ISO.

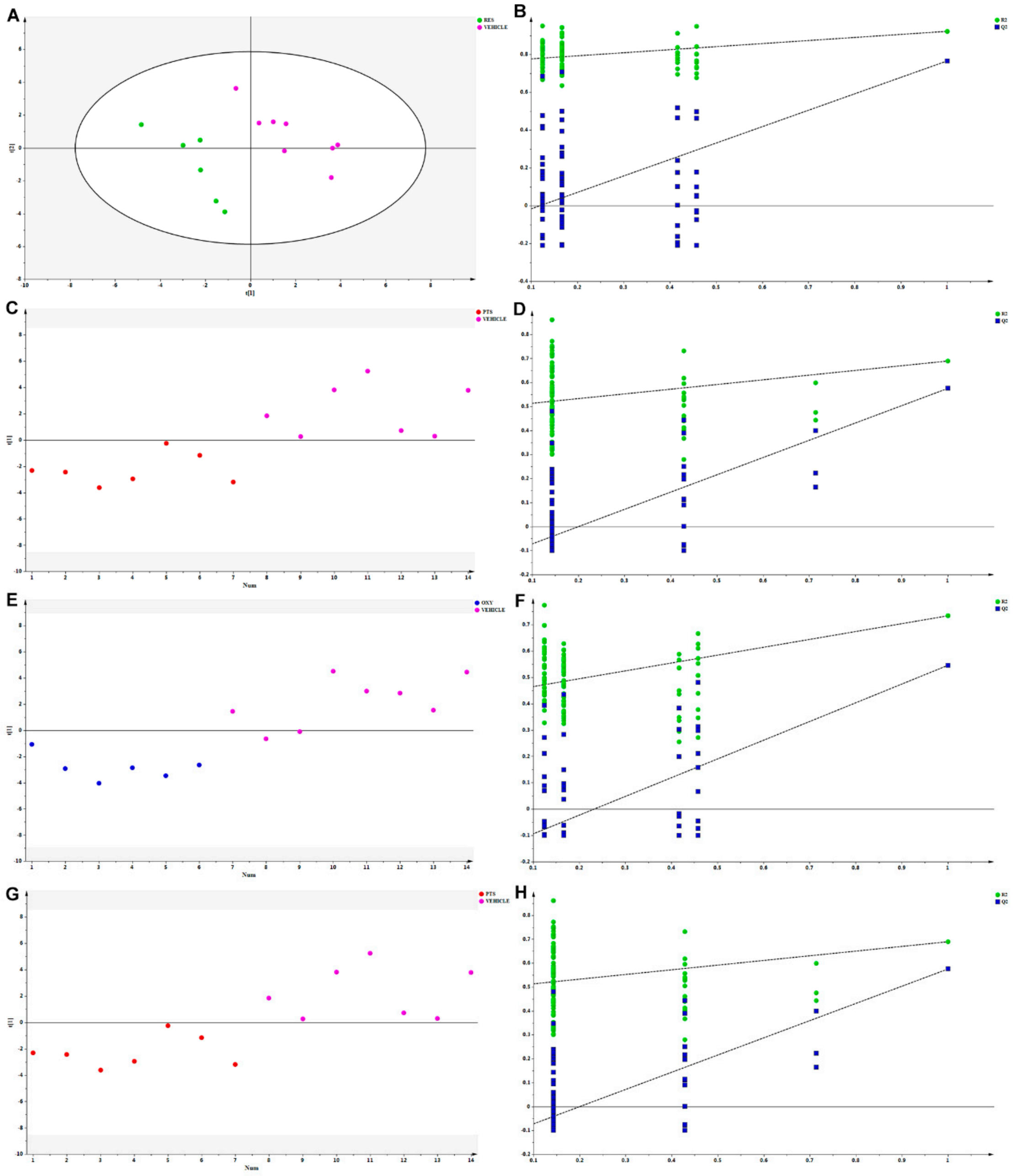

Supplementary Figure S3. PLS-DA scores plot comparing (A) RES, (C) PTS, (E) OXY and (G) ISO with vehicle in plasma. Permutation graphs for PLS-DA model in plasma samples: (B) RES, (D) PTS, (F) OXY and (H) ISO.

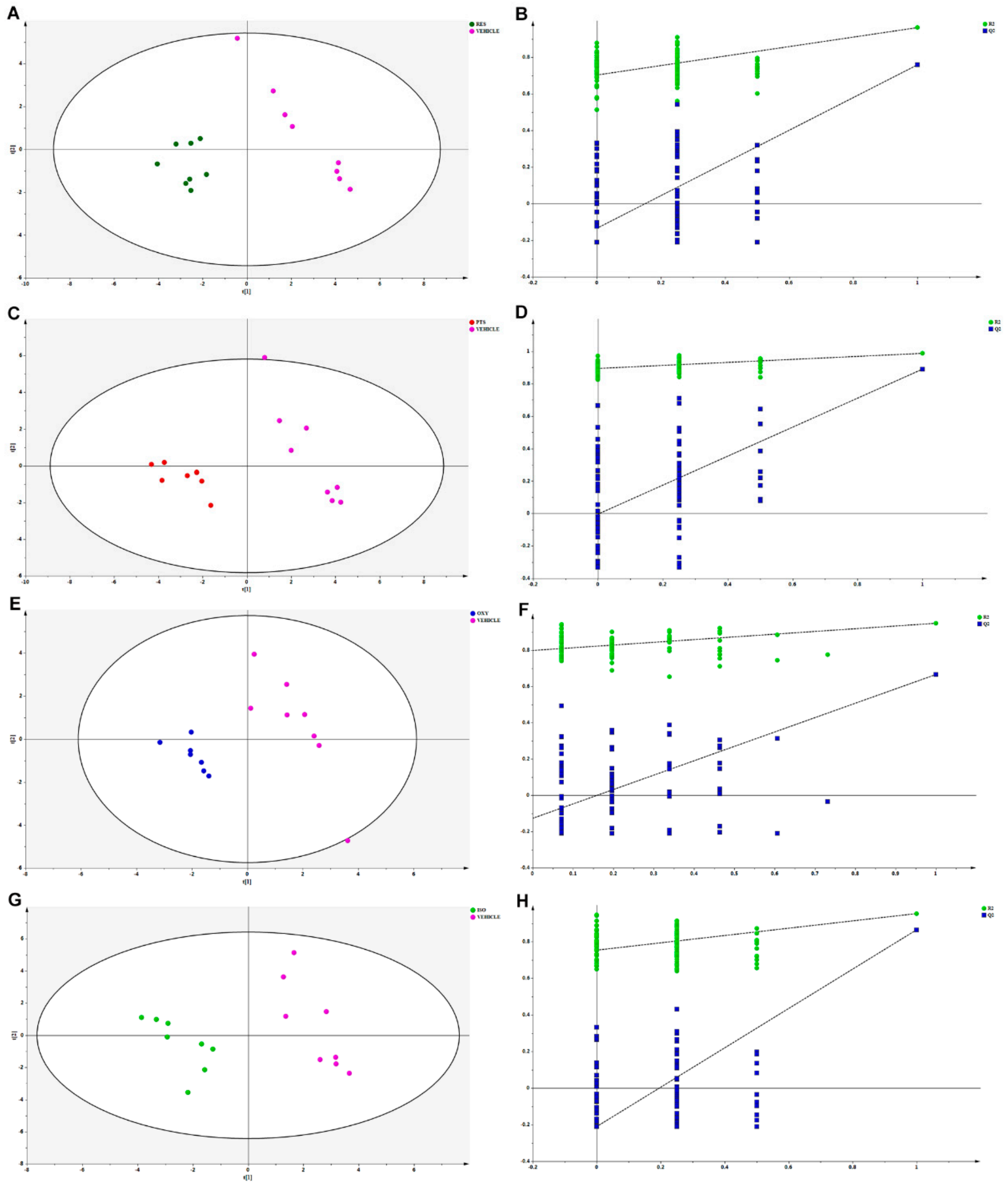

Supplement: Supplementary file 1 [file ijms-25-11027-s001.zip › ijms-3247664-supplementary.pdf]
